# Nano‐Plumber Reshapes Glymphatic‐Lymphatic System to Sustain Microenvironment Homeostasis and Improve Long‐Term Prognosis after Traumatic Brain Injury

**DOI:** 10.1002/advs.202304284

**Published:** 2023-10-22

**Authors:** Shiqiang Tong, Laozhi Xie, Xiaoying Xie, Jianpei Xu, Yang You, Yinzhe Sun, Songlei Zhou, Chuchu Ma, Gan Jiang, Fenfen Ma, Zhihua Wang, Xiaoling Gao, Jun Chen

**Affiliations:** ^1^ Department of Pharmaceutics School of Pharmacy & Shanghai Pudong Hospital Fudan University Shanghai 201203 China; ^2^ Key Laboratory of Smart Drug Delivery Ministry of Education School of Pharmacy Fudan University Shanghai 201203 China; ^3^ Department of Pharmacology and Chemical Biology State Key Laboratory of Oncogenes and Related Genes Shanghai Universities Collaborative Innovation Center for Translational Medicine Shanghai Jiao Tong University School of Medicine Shanghai 200025 China; ^4^ Department of Pharmacy Shanghai Pudong Hospital Fudan University Shanghai 201399 China; ^5^ Department of Emergency Shanghai Pudong Hospital Fudan University Pudong Medical Center Shanghai 201399 China

**Keywords:** brain homeostasis, drug delivery system, gene transfection, nanotechnology, tissue regeneration and repair, traumatic brain injury

## Abstract

Traumatic brain injury (TBI) is a leading cause of death and disability worldwide. Long‐term changes in the microenvironment of the brain contribute to the degeneration of neurological function following TBI. However, current research focuses primarily on short‐term modulation during the early phases of TBI, not on the critical significance of long‐term homeostasis in the brain microenvironment. Notably, dysfunction of the glymphatic‐lymphatic system results in the accumulation of danger/damage‐associated molecular patterns (DAMPs) in the brain, which is regarded as the leading cause of long‐term microenvironmental disturbances following TBI. Here, a nanostructure, Nano‐plumber, that co‐encapsulates the microenvironment regulator pro‐DHA and the lymphatic‐specific growth factor VEGF‐C is developed, allowing for a sustainable and orderly regulation of the microenvironment to promote long‐term neurological recovery. Nano‐plumber reverses the injury microenvironment by suppressing microglia and astrocytes activation and maintaining reduced activation via enhanced glymphatic‐lymphatic drainage, and significantly improves the neurological function of rodents with TBI. This study demonstrates that glymphatic‐lymphatic system reconstruction is essential for enhancing long‐term prognosis following TBI, and that the Nano‐plumber developed here may serve as a clinically translatable treatment option for TBI.

## Graphical abstract

A co‐delivery system, Nano‐plumber, co‐encapsulates the microenvironment regulator pro‐DHA and the lymphatic‐specific growth factor VEGF‐C. It enables rapid reversal of injury microenvironment and maintains the homeostasis of the reversed microenvironment via enhanced glymphatic‐lymphatic drainage for long‐term neurological recovery. Consequently, Nano‐plumber provides a potential strategy for TBI treatment and offers valuable insights into the treatment of other neurological disorders.

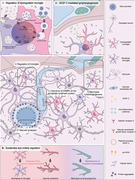

## 1 Introduction

Traumatic brain injury (TBI), caused by destructive external forces, affects nearly 60 million people worldwide each year.^[^ ^1^ ^]^ TBI causes permanent cognitive, motor, and sensory deficits, as well as frequent complications.^[^ ^2^ ^]^ Despite considerable research efforts, there remains a dearth of effective therapeutic agents and interventions capable of promoting neurological recovery and improving patient prognosis.

TBI involves both primary and secondary injuries. The former arises from direct mechanical forces acting on the brain, leading to immediate tissue damage, while the latter is characterized by a cascade of cellular and biochemical changes, including inflammation, oxidative stress, changes in blood‐brain barrier (BBB) permeability, and mitochondrial dysfunction.^[^ ^3^ ^]^ The secondary injury sustained in TBI can persist over the long term under intricate pathological mechanisms, gradually evolving into permanent brain disorder and triggering the development of associated complications such as Alzheimer's disease (AD), Parkinson's disease (PD), and epilepsy.^[^ ^2^, ^4^ ^]^ Recently, studies have focused on the development of anti‐inflammatory and antioxidant strategies targeting the early stages of TBI, aiming to mitigate neuronal loss and alleviate neurological dysfunction following TBI.^[^ ^5^ ^]^ Despite that these existing strategies can temporarily suppress inflammation levels, they are unable to prevent the recurrence of complex pathological imbalances in the lesion microenvironment after drug depletion, making it challenging to overcome the long‐term chronic damage that restricts patient prognosis.^[^ ^6^ ^]^ Consequently, an optimal strategy for neurological recovery after TBI should encompass the entire injury course and be capable of sustainably regulating the microenvironment at the lesion site.

The glymphatic‐lymphatic system, comprised primarily of meningeal lymphatic vessels (MLVs) and perivascular spaces (PVS),^[^ ^7^ ^]^ plays a vital role in maintaining the dynamic homeostasis of the brain environment by clearing danger/damage‐associated molecular patterns (DAMPs) in a general and non‐selective manner.^[^ ^8^ ^]^ Cerebral interstitial fluid (ISF), which contains molecules derived from the central nervous system and neurotoxic substances, enters the subarachnoid space via the PVS and is then evacuated by MLVs to the deep cervical lymph nodes (dCLN).^[^ ^7^, ^9^ ^]^ The primary and secondary injuries in TBI give rise to a substantial build‐up of inflammatory DAMPs, and the expeditious clearance of these DAMPs is contingent upon the proper functioning of the glymphatic‐lymphatic system. However, TBI induces long‐term drainage impairment of MLVs and severe dysfunction of central blood vessels,^[^ ^10^ ^]^ impeding efflux of DAMPs released from injured brain cells.^[^ ^4^, ^8^ ^]^ The accumulated DAMPs stimulate local glial cells and infiltrating immune cells to secrete large amounts of pro‐inflammatory cytokines, including tumor necrosis factor‐α (TNF‐α), interleukin‐6 (IL‐6), and interleukin‐1β (IL‐1β), as well as increasing levels of reactive oxygen species (ROS), leading to ongoing damage to surrounding normal neurons. This cycle results in further release of DAMPs and ROS, perpetuating sustained and malignant neuroinflammation and long‐term disruption of the brain microenvironment following TBI.^[^ ^7^, ^9^ ^]^ Consequently, the impairment of the glymphatic‐lymphatic system function after TBI is considered to be the primary cause of persistent neurological dysfunction and frequent complications, and restoring its drainage function may be a crucial step in sustainably regulating the brain microenvironment to improve the long‐term prognosis after TBI.

To reshape the glymphatic‐lymphatic system and restore its persistent regulation of the lesion microenvironment following TBI, we propose a nano‐drug delivery system that promotes neovascularization of meningeal lymphatic vessels. Vascular endothelial growth factor C (VEGF‐C), a lymphatic vessel‐specific growth factor, promotes the proliferation and migration of lymphatic endothelial cells and induces the peripheral blood endothelial progenitor cells migrating to the injured brain to differentiate into lymphatic endothelial cells, thereby promoting lymphangiogenesis.^[^ ^11^ ^]^ VEGF‐C can be applied to promote the meningeal lymphangiogenesis to enhance lymphatic drainage capacity and regain homeostasis in the brain injury microenvironment. However, cytokine‐based therapy is restricted by their short half‐life, substantial side effects, and poor BBB penetration ability after systemic administration. To enhance the delivery of VEGF‐C in vivo, we chose to encapsulate a plasmid encoding VEGF‐C (pVEGFC) with lipid calcium phosphate (CaP) technology.^[^ ^12^ ^]^ Moreover, because VEGF‐C‐mediated meningeal lymphangiogenesis is a time‐consuming process,^[^ ^11^ ^]^ rapid reversal of the brain injury microenvironment is required to mitigate neurological damage during this time. The enzyme histone deacetylase (HDAC) plays a crucial role in the aberrant activation of microglia, which promotes abnormalities in phagocytic, inflammatory, and oxidative stress‐related pathways.^[^ ^13^ ^]^ Our preliminary studies found that propofol‐docosahexaenoic acid (pro‐DHA),^[^ ^14^ ^]^ a self‐synthesized HDAC inhibitor that is less cytotoxic than other HDAC inhibitors, can effectively reverses the inflammatory phenotype and phagocytosis dysfunction of dysregulated microglia. Given that the PVS is a crucial component of the glymphatic‐lymphatic system,^[^ ^7^ ^]^ it is essential to preserve vascular integrity after TBI. Excitingly, pro‐DHA has demonstrated promising potential for vascular integrity protection. Thus, we co‐deliver pro‐DHA in the pVEGFC‐encapsulated nanostructure to obtain dual‐delivery system pro‐DHA/pVEGFC@CaP, thereby modulating the disordered microenvironment caused by dysregulated microglia and restoring central vascular function after TBI. Galectin‐3 (Gal‐3), a member of galectins family, is upregulated on dysregulated microglia following TBI.^[^ ^15^ ^]^ Galactose (Gal) is modified on pro‐DHA/pVEGFC@CaP to obtain Gal‐pro‐DHA/pVEGFC@CaP to enhance its dysregulated microglia targeting selectivity by binding with Gal‐3.

**Figure 1:**
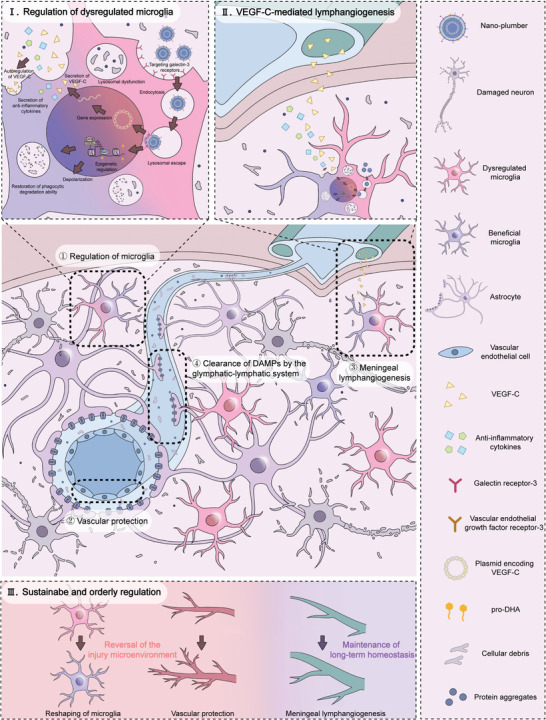
Nano‐plumber reshapes glymphatic‐lymphatic system to sustain microenvironment homeostasis and improve long‐term prognosis after traumatic brain injury. The functions of Nano‐plumber can be classified into the following aspects: ①Nano‐plumber modulates dysregulated microglia and reverses injury microenvironment prior to the remodeling of meningeal lymphatic vessels (refer to enlarged Figure I); ②Nano‐plumber protects vascular to maintain the function of PVS; ③Nano‐plumber promotes the meningeal lymphangiogenesis (refer to enlarged Figure II);④Enhanced drainage capacity of meningeal lymphatic vessels coupled with perivascular drainage pathways for central DAMPs enables long‐term maintenance of injury microenvironment after TBI. Based on these aspects, Nano‐plumber can regulate injury microenvironment sustainably and orderly following TBI (refer to Figure III).

Collectively, the design of Gal‐pro‐DHA/pVEGFC@CaP takes into account the following factors: pro‐DHA increases the phagocytic activity of microglia and the transfection efficiency of pVEGFC in orthotopic TBI, promoting the reshape of the glymphatic‐lymphatic system; The co‐delivery system facilitates the rapid reversal and maintenance of the brain injury microenvironment throughout the course of TBI; In comparison to conventional viral vectors, the advantages of Gal‐pro‐DHA/pVEGFC@CaP are that its transfection toxicity to normal cells can be reduced due to its selectivity of dysregulated microglia cells, and its transient transfection can be controlled to prevent brain immune activation induced by overgrowth of meningeal lymphatic vessels.^[^ ^11^, ^16^ ^]^ Due to the capacity of Gal‐pro‐DHA/pVEGFC@CaP to reshape the drainage functions of meningeal lymphatic vessels and blood vessels, we name it as “Nano‐plumber”. **Figure** **1** depicts the potential therapeutic efficacy of Nano‐plumber. Our design is anticipated to accomplish orderly and long‐term regulation of regeneration and repair after TBI, thereby providing a potential strategy for TBI treatment and offering valuable insights into the treatment of other neurological disorders.

## 2 Results

### 2.1 The Preparation and Characterization of Nano‐Plumber

**Figure 2:**
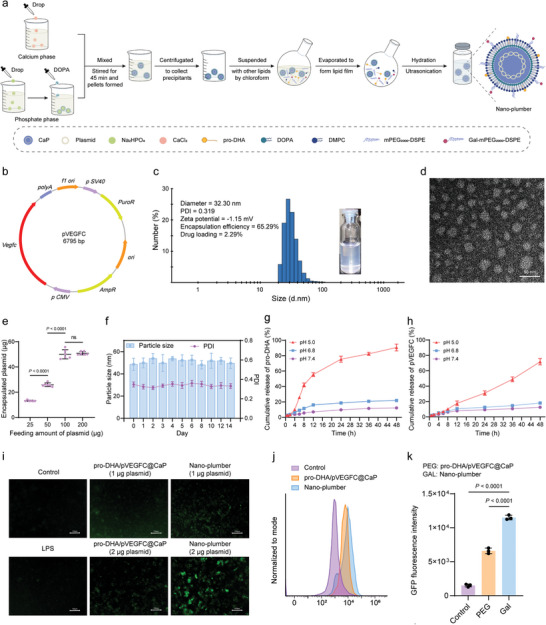
The preparation and characterization of Nano‐plumber. a) Scheme for the preparation of Nano‐plumber. b) Plasmid encoding VEGF‐C. c) Appearance and size distribution of Nano‐plumber detected by DLS. d) TEM of Nano‐plumber. e) The amount of plasmid encapsulated at various feeding levels (*n* = 6). f) Stability of Nano‐plumber in 5% Glu (*n* = 3). g,h) The release profile of pro‐DHA (g) and pVEGFC (h) from Nano‐plumber in PBS (pH 5.0, pH 6.5 and pH 7.4) at 37 °C (*n* = 3). i) Representative images of BV2 cells transfected with EGFP‐PEG‐pro‐DHA/pVEGFC@CaP or EGFP‐Nano‐plumber. j, k) Representative histogram j) and quantification k) of flow cytometry analysis on BV2 cells transfected with EGFP‐PEG‐pro‐DHA/pVEGFC@CaP or EGFP‐Nano‐plumber (*n* = 3). Data represent mean ± SD of three independent biological replicates. Statistical analyses are performed using one‐way ANOVA with Tukey's post hoc test.

Nano‐plumber was prepared using the reversed‐phase microemulsion method and thin film dispersion technique. The HDAC inhibitor pro‐DHA is a compound consisting of a conjugation between propofol and docosahexaenoic acid (DHA), synthesized by a typical esterification reaction. The synthetic procedure was conducted following the previously established method (Figure S1, Supporting Information).^[^ ^14^ ^]^ The preparation process of Nano‐plumber is illustrated in **Figure** **2**a. In brief, a plasmid encoding the VEGF‐C (pVEGFC) sequence was constructed (Figure 2b; Figure S4b, Supporting Information). Next, a drug‐loaded calcium phosphate core was synthesized via the reversed‐phase microemulsion method.^[^ ^17^ ^]^ The resulting precipitate was then combined with additional lipids and pro‐DHA in chloroform to form a thin film under high vacuum. Subsequently, a 5% glucose (Glu) solution was added to hydrate the lipid film, resulting in the formation of Nano‐plumber. Gal‐pro‐DHA@CaP(without pVEGFC), Gal‐pVEGFC@CaP(without pro‐DHA), and PEG‐pro‐DHA/pVEGFC@CaP(without Galactose modification) were also synthesized using identical procedure.

The particle size of Nano‐plumber was determined to be approximately 32 nm or 30 nm by dynamic light scattering detector (DLS) or transmission electron microscope (TEM), respectively (Figure 2c,d). The surface charge was approximately −1.15 mV (Figure 2c), and the encapsulation efficiency (EE) of plasmid and pro‐DHA were 50.82 ± 4.68% and 65.29 ± 5.25%, respectively (Figure 2c,e; Figure S2, Supporting Information). The loading efficiency (LE) of plasmid and pro‐DHA were determined to be 0.22 ± 0.02% and 2.29 ± 0.21%, respectively (Figure 2c,e; Figure S2, Supporting Information). Nano‐plumber exhibited good stability (Figure 2f; Figure S3, Supporting Information). The two drugs released slowly from Nano‐plumber at pH 7.4 (mimicking phyiological circumstances) and pH 6.8 (mimicking brain injury microenvironment), but the release rate rose dramatically at pH 5.0 (mimicking lysosomes; Figure 2g,h), indicating that the release of the encapsulated drugs was pH‐dependent.

To evaluate the transfection efficiency of Nano‐plumber, we constructed EGFP‐ Nano‐plumber using a plasmid encoding both enhanced green fluorescent protein (EGFP) and VEGF‐C sequences (Figure S4a, Supporting Information). Inverted fluorescence imaging revealed a significantly transfection efficiency in BV2 cells pre‐stimulated with lipopolysaccharide (LPS) (Figure 2i). Flow cytometry was applied to quantitatively analyze the transfection efficiency of EGFP‐Nano‐plumber after transfection with BV2 cells for 48 h. The transfection efficiency of EGFP‐Nano‐plumber was found to be 1.74 times higher than that of EGFP‐PEG‐pro‐DHA/pVEGFC@CaP (Figure 2j,k), indicating that the lactose modification facilitates uptake by microglial to improve transfection efficiency.

### 2.2 Nano‐Plumber Targeted Dysregulated Microglia in the Injured Brain Region

**Figure 3:**
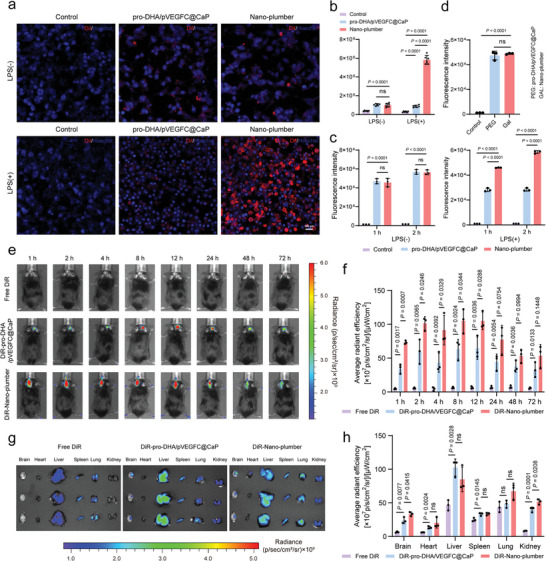
Nano‐plumber targeted dysregulated microglia in the injured brain region. a) Cell uptake of Nano‐plumber labeling with DiI. Images were representative of three experiments. b) Semi‐quantitative results of cellular uptake of Nano‐plumber using fluorescence (*n* = 3). c) Flow cytometry analysis of cell uptake of Nano‐plumber (*n* = 3). d) Flow cytometric analysis of uptake inhibition of Nano‐plumber. Blockade experiment was performed by pretreating activated BV2 cells with free galactose (100 µg mL^−1^) for 15 min before Nano‐plumber incubation (*n* = 3). e) Real‐time whole‐body DiR fluorescence imaging of CCI mice after *i.v*. injection of free DiR, DiR‐labeled PEG‐pro‐DHA/pVEGFC@CaP or DiR‐labeled Nano‐plumber. f) Semi‐quantitative results of the in vivo brain radiant efficiency shown in (e) (*n* = 3). g) Ex vivo DiR fluorescence imaging of main organs obtained from CCI mice at 72 h post‐injection. h) Semi‐quantitative results of the *ex vivo* main organs radiant efficiency shown in (g) (*n* = 3). Data represent mean ± SD of three independent biological replicates. Statistical analyses are performed using one‐way ANOVA with Tukey's post hoc test.

The modification of lactose confers Nano‐plumber with the capability of targeting dysregulated microglial.^[^ ^15^, ^18^ ^]^ To verify the targeting specificity of Nano‐plumber toward activated microglia, we employed a fluorescent probe DiI to label the carrier. Confocal microscopy analysis showed that the uptake of Nano‐plumber in BV2 cells pre‐stimulated with 100 ng mL^−1^ LPS was 6.7 times higher than that of PEG‐pro‐DHA/pVEGFC@CaP (**Figure** **3**a,b). Flow cytometry analysis demonstrated that after co‐incubation with BV2 cells for 1 h, the uptake of Nano‐plumber in BV2 cells was 1.63 times higher than that of PEG‐pro‐DHA/pVEGFC@CaP. Similarly, after co‐incubation with BV2 cells for 2 h, the uptake of Nano‐plumber by BV2 cells was 2.06 times higher than that of PEG‐pro‐DHA/pVEGFC@CaP (Figure 3c). Pre‐treatment of activated BV2 cells with lactose effectively inhibited the targeting of Nano‐plumber, indicating that the specificity of Nano‐plumber in selectively targeting specific cells may be achieved through specific binding to lactose‐carrying galectin‐3 receptors upregulated on the surface of activated BV2 cells (Figure 3d; Figure S5, Supporting Information).

In general, the BBB is severely compromised following TBI,^[^ ^3^ ^]^ creating a favorable environment for the penetration of Nano‐plumber at the nanoscale, which facilitates targeted drug delivery to the damaged brain tissue.^[^ ^19^ ^]^ To evaluate their potential for targeted brain tissue injury, Nano‐plumber and pro‐DHA/pVEGFC@CaP, both labeled with DiR, or free DiR were injected into controlled cortical impact (CCI) model mice. The accumulation of Nano‐plumber in the injured brain was greater than that of pro‐DHA/pVEGFC@CaP and significantly higher than free DiR from 1 to 72 h after injection (Figure 3e,f). This finding was confirmed by in vitro imaging at 72 h after injection. The accumulation of Nano‐plumber in the mice brains was 1.39 times higher than that of PEG‐pro‐DHA/pVEGFC@CaP (Figure 3g,h). The higher accumulation of Nano‐plumber at the site of brain injury may be attributed to a higher uptake of Nano‐plumber by dysregulated microglia at the site of injury.^[^ ^15^, ^18^ ^]^ Altogether, these results revealed that galactose modification facilitated the targeted delivery of Nano‐plumber to activated microglia cells and injured brain tissue.

### 2.3 Nano‐Plumber Efficiently Reshaped Dysregulated Microglia In Vitro

**Figure 4:**
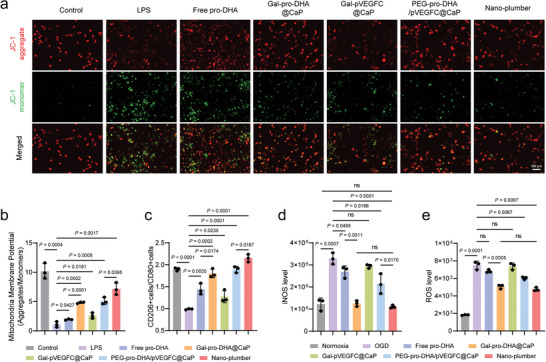
Nano‐plumber efficiently reshaped dysregulated microglia in vitro. a,b) Representative images a) and semi‐quantitative b) results of JC‐1 staining of activated BV2 cells with different treatments (*n* = 3). c) Flow cytometry analysis of CD80^+^ and CD206^+^ cells in activated BV2 cells with different treatments (*n* = 3). d,e) Quantification of the level of (d) iNOS and e) ROS in activated BV2 cells with different treatments (*n* = 3). Data represent mean ± SD of three independent biological replicates. Statistical analyses are performed using one‐way ANOVA with Tukey's post hoc test.

Microglia‐mediated inflammatory cascades following TBI contribute to neuronal damage, highlighting the significance of effective intervention targeting dysregulated microglia for rapidly reshaping the injury microenvironment.^[^ ^4^, ^20^ ^]^ Mitochondrial dysfunction is considered a crucial event in the dysregulation of microglia following TBI.^[^ ^21^ ^]^ To demonstrate the protective effect of Nano‐plumber on mitochondrial function in dysregulated microglia, we co‐cultured Nano‐plumber and its control formulation with dysregulated microglia and assessed mitochondrial membrane potential using the JC‐1 probe. JC‐1 accumulates in a potential‐dependent manner within mitochondria, existing as a monomer at low concentrations and emitting green fluorescence at ≈529 nm, and forming aggregates at higher concentrations and emitting red fluorescence at ≈590 nm. A decrease in the ratio of red to green fluorescence intensity represents a decrease in mitochondrial membrane potential, indicating impaired mitochondrial function. The results showed that the red/green fluorescence intensity ratio of normal BV2 cells was 10.17, which significantly decreased to 1.08 in BV2 cells pre‐stimulated with 100 ng mL^−1^ LPS, indicating significant mitochondrial dysfunction. Co‐incubation with Nano‐plumber led to the most significant increase in the red/green fluorescence intensity ratio, indicating the strongest protective effect of Nano‐plumber on BV2 mitochondrial function compared with other groups. The stronger protective effect of Nano‐plumber on BV2 mitochondrial function compared to PEG‐pro‐DHA/pVEGFC@CaP may be attributed to the selective accumulation of Nano‐plumber in dysfunctional microglia. Notably, the protective effect of Nano‐plumber on mitochondrial function mainly stems from the regulatory effect of pro‐DHA, and partly from the VEGF‐C expressed by cell transfection (**Figure** **4**a,b), which is consistent with previous reports.^[^ ^22^ ^]^

Microglial mitochondrial dysfunction is associated with inflammation activation, oxidative stress, and impaired phagocytic function.^[^ ^21c,d^ ^]^ Thus, we investigated the ability of Nano‐plumber to regulate the inflammatory phenotype, oxidative stress, and phagocytic function of dysregulated microglia. It was found that the ratio of M2‐like BV2 cells (CD206^+^ cells) to M1‐like BV2 cells (CD80^+^ cells) decreased from 1.89 to 0.98 after stimulation with 100 ng mL^−1^ LPS. After 24 h of co‐incubation with Nano‐plumber, the ratio of CD206^+^ BV2 cells to CD80^+^ BV2 cells significantly increased to 2.15, indicating successful reversal of the microglial inflammatory phenotype (Figure 4c; Figure S6, Supporting Information). To determine the ability of Nano‐plumber to regulate microglial oxidative stress, the levels of inducible nitric oxide synthase (iNOS) and intracellular reactive oxygen species (ROS) were measured. The results showed that Nano‐plumber significantly reduced the levels of iNOS and ROS in BV2 cells compared to the other groups (Figure 4d,e). Furthermore, restoration of microglial phagocytic clearance ability is crucial for improving the acute phase injury microenvironment.^[^ ^23^ ^]^ Therefore, we conducted a cell phagocytosis experiment and confirmed that Nano‐plumber has a significant restorative effect on the phagocytic ability of dysfunctional BV2 cells (Figure S7, Supporting Information). Taken together, these results indicate that Nano‐plumber efficiently reshaped dysregulated microglia in vitro, providing evidence for its potential role in rapid reversal of the injury microenvironment in vivo.

### 2.4 Nano‐Plumber Protected the Vascular Integrity

**Figure 5:**
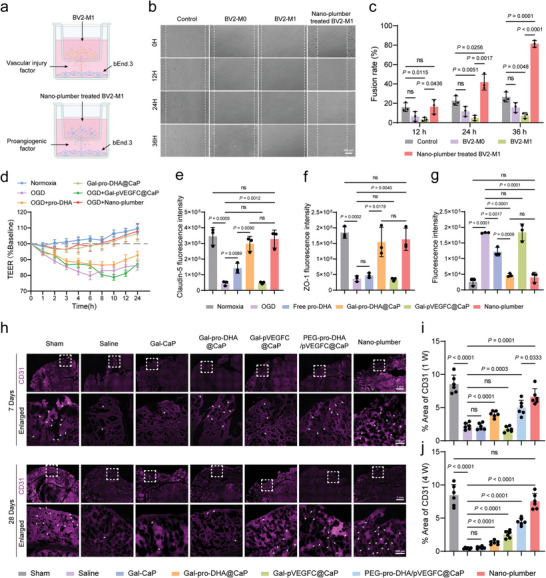
Nano‐plumber protected the vascular integrity. a) Diagram of BV2 cells with different treatments and bEnd.3 cells co‐cultured in a transwell dish. b) Brightfield images of the wound healing process of bEnd.3 in basolateral well after different treatments. c) Quantification analysis of bEnd.3 fusion rate (*n* = 3). d) Measurement of electrical resistance changes in bEnd.3 cell monolayers with different treatments (*n* = 3). e,f) Quantification of the expression of Claudin‐5 (e) and ZO‐1 (f) on bEnd.3 cells with different treatments (*n* = 3). g) Quantification of the level of ROS in bEnd.3 cells with different treatments (*n* = 3). h) Representative images of vascular endothelial cells (CD31) of various groups at 7 days and 28 days after injury (*n* = 6). i,j) Quantitative analysis showing the fluorescence intensity of various groups at 7 days (i) and 28 days (j) after injury (*n* = 6). Data represent mean ± SD of three independent biological replicates. Statistical analyses are performed using one‐way ANOVA with Tukey's post hoc test.

After a TBI, the BBB is significantly compromised, resulting in alterations in BBB permeability and exacerbating the poor condition of the injury microenvironment, which promotes neurological deterioration.^[^ ^3^ ^]^ In addition, vascular injury following TBI can impair the drainage efficacy of DAMPs from the PVS, which is detrimental to the long‐term maintenance of homeostasis in the CNS microenvironment.^[^ ^7c^ ^]^ Thus, preserving vascular integrity is of paramount importance. To assess the cerebrovascular protection potential of Nano‐plumber, we performed wound‐healing assays on bEnd.3 cells. The Nano‐plumber‐treated BV2‐M1 group exhibited the fastest bEnd.3 fusion rate, compared to the control BV2‐M0 group and BV2‐M1 group. This finding highlights the excellent cerebrovascular repair ability of Nano‐plumber treatment in BV2‐M1, which can be mainly attributed to its capacity to reverse microglial phenotype and promote the secretion of certain cytokines that are conducive to vascular healing (**Figure** **5**a–c). In addition to its indirect effect of promoting vascular recovery, we also investigated the direct protective effect of Nano‐plumber on blood vessels. The oxygen‐glucose deprivation/reoxygenation (OGD/R) treatment significantly decreased the cell resistance of the in vitro BBB model. However, compared to other formulations, the Nano‐plumber group effectively reversed the trend of significant decrease in cell resistance (Figure 5d). Moreover, treatment with Nano‐plumber significantly upregulated the expression of Claudin‐5, a protein associated with BBB integrity, by 7.22‐fold in bEnd.3 cells compared to the OGD group (Figure 5e; Figure S8, Supporting Information). Similarly, the expression of ZO‐1 in bEnd.3 was also significantly increased by 4.42‐fold after treatment with Nano‐plumber compared to the OGD group (Figure 5f; Figure S9, Supporting Information). Additionally, treatment with Nano‐plumber significantly decreased the level of ROS in bEnd.3 cells (Figure 5g). In fact, the direct effect of Nano‐plumber in promoting vascular recovery is primarily attributed to the effects of pro‐DHA on the survival, proliferation, and migration of endothelial cells, while the nano‐carrier plays a crucial role in enhancing the therapeutic effects of pro‐DHA (Figures S10–S14, Supporting Information). To summarize, Nano‐plumber can facilitate blood vessel recovery through both direct and indirect mechanisms in vitro.

To investigate the sustained protective effect of Nano‐plumber on the TBI‐injured brain vasculature in vivo, we utilized fluorescence confocal imaging to characterize the brain vasculature at 7 and 28 days post‐injury. Our findings revealed that 7 days after brain injury, the percentage of CD31 fluorescence area in the brain vasculature of mice in the saline group decreased to 25.88% of that in the sham group, indicating severe BBB disruption caused by acute injury. However, after treatment with Nano‐plumber, the percentage of CD31 fluorescence area in the injury site significantly increased to 78% of that in the Sham group. This improvement was primarily attributed to the regulatory effect of pro‐DHA in Nano‐plumber on the injury microenvironment mediated by microglia, as well as its direct protective effect on endothelial cells (Figure 5h,i).

It is noteworthy that 28 days after brain injury, the percentage of CD31 fluorescence area in the brain vasculature of mice in the saline group remained only 0.43%, suggesting prolonged inadequate restoration of BBB disruption following TBI, possibly due to re‐disruption of the injury microenvironment. Treatment with Nano‐plumber, PEG‐pro‐DHA/pVEGFC@CaP, Gal‐pVEGFC@CaP, and Gal‐pro‐DHA@CaP all improved brain vasculature levels in the injury site 28 days after TBI to some extent. Among these treatments, the Nano‐plumber group exhibited the most notable impact, elevating the percentage of CD31 fluorescence area in the injury site of mice to 17.5 times that of the saline group, surpassing the 10.39 times improvement observed in the PEG‐pro‐DHA/pVEGFC@CaP group. This improvement was mainly attributed to the lactose modification enhancing the targeting ability of the carrier to activated microglial and improving its ability to reverse rapidly and continuously maintain the microenvironment. Furthermore, the vascular protective effect of the Gal‐pVEGFC@CaP and Gal‐pro‐DHA@CaP groups was inferior to that of the Nano‐plumber group, indicating that the regulatory effect of pro‐DHA and the stabilizing effect of VEGF‐C exhibit a synergistic effect, which maximizes the recovery of brain vasculature function (Figure 5h,j). In conclusion, Nano‐plumber can provide sustained protection of brain vasculature following TBI, thereby facilitating the maintenance of a favorable brain microenvironment mediated by PVS.

### 2.5 Nano‐Plumber Rapidly Reversed the Injury Microenvironment

To achieve effective plasmid transfection in the acute phase in vivo, we first investigated the optimal dosing frequency and dosage. We administered Nano‐plumber to C57BL/6 mice established TBI models via tail vein injection using various dosing regimens. We analyzed the transfection efficiency in the brain and monitored changes in mouse body weight 24 hours after the final administration to assess the safety of each dosing regimen (Figures S15–S17, Supporting Information). Based on the experimental results, we adopted the dosing schedule of administering Nano‐plumber containing 50 µg pVEGFC to C57BL/6 mice on post‐injury day 1, 3, and 5 for subsequent experiments (Figure S18, Supporting Information).

**Figure 6:**
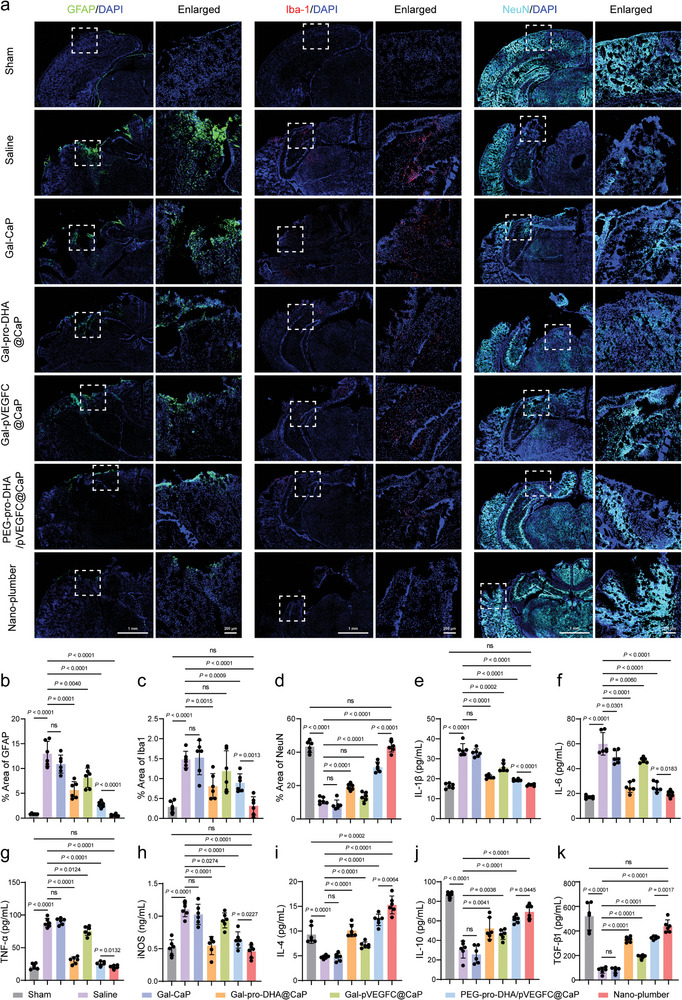
Nano‐plumber rapidly reversed the injury microenvironment. a) Representative images of glial scars (GFAP), microglia (Iba1), and neurons (NeuN) in injured sites of various groups at 7 days after injury. b–d) Quantitative analysis showing the fluorescence intensity of b) GFAP, c) Iba‐1, and d) NeuN corresponding to (a) (*n* = 3). e–h) Detection of pro‐inflammatory cytokines, such as e) IL‐1β, f) IL‐6, g) TNF‐α, and h) iNOS, in the homogenate of the injured brain of various groups at 7 days after injury (*n* = 6). i–k) Detection of anti‐inflammatory cytokines, such as h) IL‐4, i) IL‐10, and j) TGF‐β1, in the homogenate of the injured brain of various groups at 7 days after injury (*n* = 6). Data represent mean ± SD of six independent biological replicates. Statistical analyses are performed using one‐way ANOVA with Tukey's post hoc test.

Next, we investigated the Nano‐plumber‐mediated rapid reversal of the injury microenvironment following TBI using the dosing regimen established in the previous experiment. Glial fibrillary acidic protein (GFAP) is a hallmark of activated astrocytes and widely used to evaluate inflammation activation and glial scar formation in brain injury tissue.^[^ ^24^ ^]^ One week after brain injury in mice, the percentage of GFAP fluorescent area in the injury site increased significantly from the normal level of 0.82% to 12.98%, indicating the significant formation of glial scar around the TBI injury site. After one week of treatment, the Nano‐plumber group showed the most significant reduction in the percentage of GFAP fluorescent area in the mouse brain compared to the other groups, which was only 4.23% of the level in the saline group (**Figure** **6**a,b). Ionized calcium binding adapter molecule 1 (Iba‐1) is a calcium‐binding protein that is specifically expressed in microglia in the central nervous system and widely used as a marker of activated microglia to evaluate the level of microglia‐mediated inflammation.^[^ ^25^ ^]^ The results indicated a significant increase in Iba‐1 levels in the mouse brain injury area, indicating massive activation of microglia after TBI. The levels of Iba‐1 in the mouse brain injury area were decreased to some extent in the PEG‐pro‐DHA/pVEGFC@CaP, Gal‐pVEGFC@CaP, and Gal‐pro‐DHA@CaP groups. In particular, the Nano‐plumber group showed a significant decrease in Iba‐1 levels in the mouse brain injury area, even reaching the level of normal brain tissue. These findings suggest that Nano‐plumber exerts a beneficial regulatory on the abnormal activation of microglia in the brain (Figure 6a,c). The dysregulation of the injury environment is closely associated with elevated levels of decreased levels of arginase‐1 (Arg‐1). To assess this, we quantitatively analyzed the levels of Arg‐1 in the mouse brain injury area using enzyme‐linked immunosorbent assay (ELISA). The results demonstrated that treatment with Nano‐plumber significantly increases the level of Arg‐1, effectively reshaping the status of microglia (Figure S19, Supporting Information). Furthermore, Nano‐plumber treatment significantly reduced the levels of pro‐inflammatory cytokines, such as IL‐1β, IL‐6, TNF‐α, and iNOS (Figure 6e–h), while significantly increasing the levels of anti‐inflammatory cytokines, such as IL‐4, IL‐10, and TGF‐β1 (Figure 6i–k), indicating that the injury microenvironment is rapidly reversed under the action of Nano‐plumber.

To assess whether Nano‐plumber‐mediated rapid improvement of the injury microenvironment could effectively alleviate neuronal damage, we measured the level of neuronal nuclei (NeuN) in the mouse brain injury area. Our findings reveal that the level of NeuN in the mouse brain injury area decreased to 25.54% of the normal level after TBI, while the Nano‐plumber treatment group displayed the least neuronal loss and maintained a level similar to the normal group (Figure 6a,d). In summary, the administration of Nano‐plumber according to the above protocol can effectively inhibit the activation of microglia and astrocytes during the early stages of TBI, thereby reversing the injury microenvironment and protecting neurons from damage.

### 2.6 Nano‐Plumber Promoted the Meningeal Lymphangiogenesis

**Figure 7:**
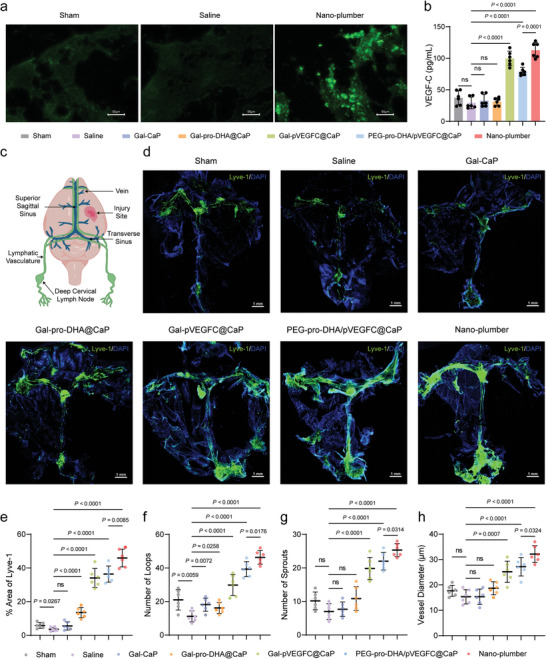
Nano‐plumber promoted the meningeal lymphangiogenesis. a) Representative images of the transfection of EGFP coding Nano‐plumber in brain injury region. b) Detection of VEGF‐C in the homogenate of the injured brain of various groups at 7 days after injury (*n* = 6). c) Spatial distribution of CNS meningeal lymphatic vessels and and location of injury site. d) Representative images of meningeal lymphatic vessels (Lyve‐1) of various groups at 28 days after injury. e) Quantification of the percent area coverage of Lyve‐1 antibody staining (*n* = 6). f,g) Quantification of the number of (f) loops and (g) sprouts in meningeal whole mounts (*n* = 6). h) Quantification of the diameters of the meningeal lymphatic vessels. Each data point represents an independent mouse and is an average of 70 measurements along the transverse and superior sagittal sinuses per mouse (*n* = 6). Data represent mean ± SD of six independent biological replicates. Statistical analyses are performed using one‐way ANOVA with Tukey's post hoc test.

After 24 hours following the last intravenous injection of Nano‐plumber, we observed significant fluorescence protein expression in the brain injury region (**Figure** **7**a; Figure S15, Supporting Information). Importantly, we observed a significant increase in VEGF‐C levels in the brains of mice in the Nano‐plumber group on day 7 after treatment, which is sufficient to effectively promote the meningeal lymphangiogenesis subsequently (Figure 7b).

Meningeal lymphatics are primarily symmetrically distributed primarily along the coronal, sagittal, and lambdoid sutures, centrally involving in the drainage of macromolecules and cellular debris from the brain to the deep cervical lymph nodes (dCLN) (Figure 7c).^[^ ^7c^ ^]^ To assess the effect of Nano‐plumber in promoting post‐TBI meningeal lymphangiogenesis, we collected mouse meninges 4 weeks after administration and observed the distribution of meningeal lymphatic vessels using fluorescence confocal microscopy. The results showed that after 4 weeks of TBI modeling, meningeal lymphatic vessels were significantly suppressed, and the percentage of the fluorescent area of the lymphatic vessel marker Lyve‐1 decreased from 5.14% to 3.35%, consistent with previous findings. Compared with other treatment groups, we observed a significant increase in the percentage of the fluorescent area of Lyve‐1 in the meninges of mice treated with Nano‐plumber after 4 weeks, reaching 47%. Importantly, the fluorescent intensity of Lyve‐1 in the meninges of mice treated with Nano‐plumber was 1.29 times higher than that in the meninges of mice treated with Nano‐plumber, suggesting that the enhanced cell‐targeting ability of Nano‐plumber may have led to increased transfection efficiency of plasmids, thereby promoting meningeal lymphangiogenesis (Figure 7d,e).

Furthermore, meningeal lymphangiogenesis is typically achieved through sprouting, looping, and an increase in vessel diameter. Compared to untreated mice, we observed a significant increase in the number of capillary loops and sprouts, as well as a two‐fold increase in the average diameter of meningeal lymphatic vessels in the Nano‐plumber group after 4 weeks (Figure 7d–h). These results demonstrated that Nano‐plumber can effectively promote lymphangiogenesis by overexpressing VEGF‐C at the injury site, thereby enhancing the drainage function of meningeal lymphatic vessels. By improving meningeal lymphatic drainage function, it can help maintain a homeostasis of brain injury microenvironment, creating a nurturing environment that promotes endogenous neural regeneration and repair.

### 2.7 Nano‐Plumber Continuously Maintained Homeostasis in a Reversed Injury Microenvironment

**Figure 8:**
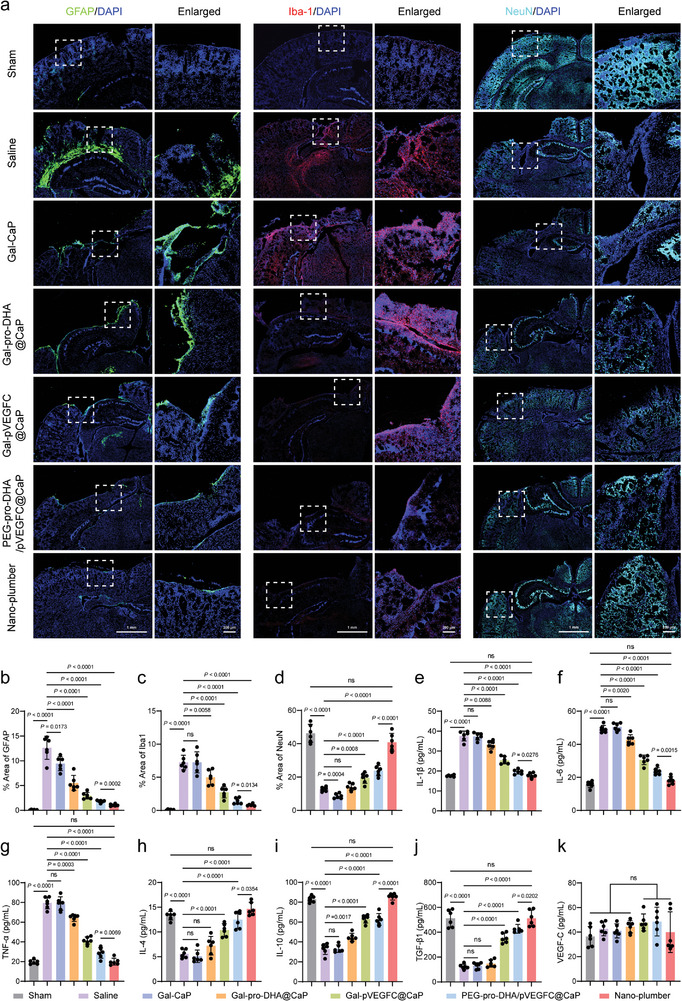
Nano‐plumber continuously maintained homeostasis in a reversed injury microenvironment. a) Representative images of glial scars (GFAP), microglia (Iba1) and neurons (NeuN) in injured sites of various groups at 28 days after injury. b–d) Quantitative analysis showing the fluorescence intensity of b) GFAP, c) Iba‐1, and d) NeuN corresponding to (a) (*n* = 3). e–g) Detection of pro‐inflammatory cytokines, such as e) IL‐1β, f) IL‐6, and g) TNF‐α, in the homogenate of the injured brain of various groups at 28 days after injury (*n* = 6). h–j) Detection of anti‐inflammatory cytokines, such as h) IL‐4, i) IL‐10, and j) TGF‐β1, in the homogenate of the injured brain of various groups at 28 days after injury (*n* = 6). k) Detection of VEGF‐C in the homogenate of the injured brain of various groups at 28 days after injury (*n* = 6). Data represent mean ± SD of six independent biological replicates. Statistical analyses are performed using one‐way ANOVA with Tukey's post hoc test.

Nano‐plumber promotes the maintenance of homeostasis in injured brain by enhancing the drainage of meningeal lymphatic vessels and maintaining normal brain vascular function. To evaluate the sustained regulatory effects of Nano‐plumber on the injury microenvironment, we performed fluorescence confocal imaging of the mouse brain injury area. As expected, despite the passage of significant time after TBI, the activation levels of astrocytes and microglia in the mouse brain injury site remained significantly elevated compared to normal levels. Nano‐plumber treatment resulted in a decrease of the GFAP fluorescent area percentage to 1.05% in the mouse brain injury site, while the Nano‐plumber group showed a decrease to 1.8%. The difference between the two groups can be mainly attributed to the higher transfection efficiency of Nano‐plumber. The inhibitory effect of Nano‐plumber on astrocyte activation was significantly greater than that of Gal‐pro‐DHA@CaP and Gal‐pVEGFC@CaP groups, indicating a synergistic effect of pro‐DHA and VEGF‐C in regulating the microenvironment at different stages (**Figure** **8**a,b). Moreover, Nano‐plumber treatment significantly decreased the activation level of microglia in the injury area 28 days after TBI (Figure 8a,c). Subsequently, the levels of iNOS and Arg‐1 in the mouse brain injury area were quantified using ELISA. The results demonstrated that the Nano‐plumber treatment group exhibited a significant decrease in iNOS levels to 50 pmol mL^−1^ and a significant increase in Arg‐1 levels to 100 pmol mL^−1^ at the site of brain injury in mice (Figure S20, Supporting Information). As a result of the effective inhibition of microglial and astrocytic activation, the Nano‐plumber treatment group showed lower levels of pro‐inflammatory cytokines such as IL‐1β, IL‐6, and TNF‐α in the mouse brain (Figure 8e–g), while levels of anti‐inflammatory cytokines such as IL‐4, IL‐10, and TGF‐β, were significantly increased in 28 days after injury (Figure 8h–j).

Based on prolonged microenvironmental modulation of Nano‐plumber after TBI, a reduction in neuronal loss and an increase in endogenous neural regeneration was observed in the mouse injury site. Compared to other treatment groups, the level of neurons in the injury site of mice treated with Nano‐plumber recovered to 88.47% of the normal group. It is noteworthy that the superior therapeutic effect of Nano‐plumber is attributed to the orderly regulatory effect of pro‐DHA and VEGF‐C. Specifically, pro‐DHA alleviated neuronal damage and protected blood vessels rapidly, while VEGF‐C sustained the reversed homeostasis of the injury microenvironment, thereby promoting optimal endogenous neural repair. Hence, the absence of either function would limit neural recovery after TBI (Figure 8a,d). In addition, prolonged exposure to high levels of VEGF‐C may result in adverse effects. Hence, we quantitatively evaluated the level of VEGF‐C in the mouse brain 28 days after TBI. The results indicated that when the drainage function of the meningeal lymphatic vessels was enhanced, the level of VEGF‐C in the mouse brain gradually declined to 39.9 pg mL^−1^, substantially mitigating the risk of side effects (Figure 8k).^[^ ^26^ ^]^

Taken together, Nano‐plumber exhibited the capacity to ameliorate the acute injurious microenvironment and sustain vascular function, while continuously maintaining homeostasis of injury microenvironment. The aforementioned comprehensive approach effectively facilitated high‐quality neural repair throughout the entire course of TBI.

### 2.8 Nano‐Plumber Facilitated Long‐Term Neurological Recovery and Mitigated Comorbid AD after TBI

**Figure 9:**
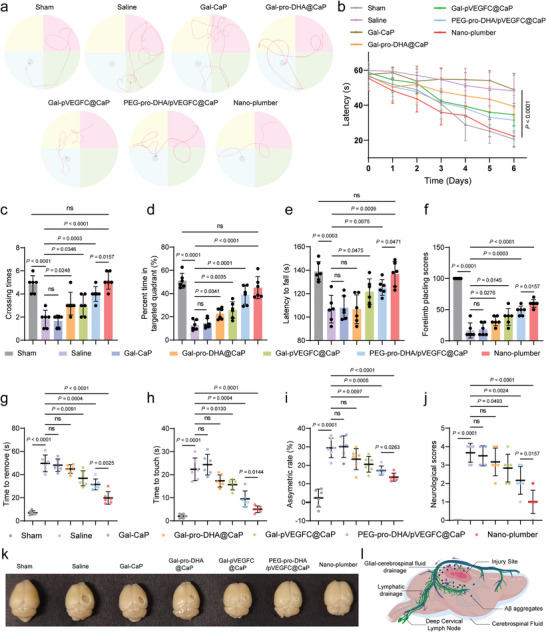
Nano‐plumber facilitated long‐term neurological recovery and mitigated comorbid AD after TBI. a) Representative swimming track of Morris water maze assays on TBI mice at 28 days after injury. b–d) Quantitative analysis of b) escape latency, c) time of crossing the platform, and d) percentage of time spent in the target quadrant based on Morris water maze assays at 28 days after injury (*n* = 6). e–i) Behavior tests including e) rotarod test, f) forelimb placing, g–h) adhesive, and i) cylinder tests were assessed at 28 days after injury (*n* = 6). j) Neurological scores of the CCI mice after treatment with different formulations (*n* = 6). k) Representative image of the ulcerated area at the site of brain injury in various groups at 28 days after injury (*n* = 6). l) The schematic diagram illustrating the alleviation of Aβ burden by Nano‐plumber. Data represent mean ± SD of six independent biological replicates. Statistical analyses are performed using one‐way ANOVA with Tukey's post hoc test.

To investigate the impact of Nano‐plumber treatment on long‐term neurological function recovery after TBI, we initially utilized the Morris water maze to assess cognitive and motor ability recovery in mice at 28 days post‐injury. The swimming paths revealed that the Nano‐plumber group demonstrated the shortest path to reach the platform in comparison to the other groups (**Figure** **9**a). Quantitative analyses revealed that the Nano‐plumber group exhibited superior long‐term neurological recovery compared to the other groups (Figure 9b–d). TBI resulted in substantial impairment of sensorimotor functions, as evidenced by shortened latency on the rotarods, reduced forelimb placing scores, prolonged time to remove and time to touch adhesive stickers, and increased asymmetric rate. In contrast, treatment with Nano‐plumber facilitated neurological recovery (Figure 9e–i). Furthermore, the neurological score of mice subjected to CCI and treated with saline was evaluated as 4.0, indicating severe neurological deficit. However, treatment with Gal‐pro‐DHA@CaP, Gal‐pVEGFC@CaP, PEG‐pro‐DHA/pVEGFC@CaP, and Nano‐plumber all improved neurologic function to varying degrees. Notably, the Nano‐plumber group exhibited the most significant improvement in neurological recovery, with the neurological score of CCI mice decreasing to 1.0 (Figure 9j). Moreover, treatment with Nano‐plumber significantly improved the ulcerated area at the site of brain injury in mice (Figure 9k). These results collectively suggest that Nano‐plumber exhibited the most significant effect on neurofunctional recovery after TBI, which can be primarily attributed to two main factors. First, the modification of the lactose targeting moiety enhanced the transfection efficiency of Nano‐plumber for activated microglia. Second, Nano‐plumber achieved comprehensive and long‐term regulation of the microenvironment in the TBI‐injured region, reducing the loss of neurons in the early stage and creating a favorable regenerative microenvironment for endogenous neurorepair.

Chronic inflammation following TBI is strongly associated with the occurrence of related complications,^[^ ^2a–e^ ^]^ such as Alzheimer's disease (AD). Excessive production of beta‐amyloid (Aβ) in injured neurons induced by chronic inflammation after TBI can accumulate in the brain parenchyma, leading to neurodegenerative diseases.^[^ ^27^ ^]^ Nano‐plumber can enhance the drainage function of glymphatic‐lymphatic system, which may help to remove excessive Aβ outside of the brain, reducing the risk of AD following TBI (Figure 9l). To investigate whether Nano‐plumber could reduce the burden of Aβ in the brain after TBI, we performed immunofluorescence analysis of mouse brain sections. Our results showed that 28 days after TBI, the percentage of Aβ fluorescence area in the injured cortex of mice increased from 0.03% to 2.08%, in the hippocampus from 0.06% to 1.75%, and in the thalamus from 0.22% to 2.87%, indicating a significant increase in Aβ burden after TBI. Compared to other treatment groups, Nano‐plumber significantly reduced the Aβ burden in the mouse brain injury area, with a significant decrease in the percentage of Aβ fluorescence area in the mouse brain cortex to 0.17%, in the hippocampus to 0.10%, and in the thalamus to 0.23% (Figure S21, Supporting Information). The above findings suggest that Nano‐plumber not only facilitated neurobehavioral recovery but also mitigates the risk of TBI‐associated AD, highlighting its significance in improving patient long‐term outcomes.

Along with neurological recovery effectiveness, safety is of paramount importance. We evaluated the cytotoxicity of Nano‐plumber by co‐incubating cells with varying concentrations (Figures S22–S24, Supporting Information). To assess the potential toxicity of various therapies in vivo, the same delivery schedule as described above was used on healthy C57BL/6 mice. No discernible structural changes were observed in the hearts, livers, spleens, lungs, or kidneys were found (Figure S25, Supporting Information), confirming the safety of the Nano‐plumber.

## 3 Conclusion

Despite extensive research into the neurological recovery following TBI, effective clinical treatments remain elusive due to the complexity of TBI pathology. Increasing understanding of TBI pathology has led to a growing focus on interventions targeting pathological pathways such as inflammation, oxidative stress, and mitochondrial dysfunction in the acute phase of TBI.^[^ ^5^ ^]^ Nerve regeneration and repair is a lengthy process, and current strategies have limited duration of action, making it challenging to maintain a stable microenvironment at the lesion site over the long term, which results in poor neurological recovery and frequent complications.^[^ ^6^ ^]^ While regulation during the early stages of TBI is critical, a comprehensive and efficient regulation strategy during this phase can protect existing neurons to the greatest extent possible, thus reducing the subsequent burden of endogenous repair. Therefore, it is necessary to develop a treatment modality that combines rapid reversal of injury microenvironment with the ability to maintain long‐term dynamic homeostasis in injured brain, covering the entire disease course, which is critical to overcoming treatment bottlenecks in TBI and minimizing complications.

This study aimed to explore a therapeutic strategy that integrates the rapid reversal of the injury microenvironment and the persistent maintenance of homeostasis in the injury microenvironment, in order to effectively promote the recovery of neurological function after TBI based on its pathological features. Our previously synthesized HDAC inhibitor,^[^ ^13^ ^]^ pro‐DHA,^[^ ^14^ ^]^ possesses the potential to comprehensively reverse the injury microenvironment by reversing the inflammatory phenotype of microglia, reshaping their phagocytic ability, and protecting blood vessels (Figures S6–S14, Supporting Information). Moreover, VEGF‐C can stimulate the neogenesis of meningeal lymphatic vessels, boost the DAMPs clearance coupled with perivascular drainage pathways, and thus achieve long‐term autonomous regulation of the brain environment.^[^ ^10^, ^11^, ^28^ ^]^ To simultaneously deliver pro‐DHA and VEGF‐C, we constructed Nano‐plumber. As expected, Nano‐plumber was effective in reshaping the injury microenvironment rapidly and sustaining the homeostasis of the injury microenvironment continuously (Figures 6 and 8; Figures S19 and S20, Supporting Information). Furthermore, behavioral experiments demonstrated that Nano‐plumber significantly improved the long‐term recovery of neurological function after TBI, primarily due to its effect of reducing neuronal loss and enhancing endogenous repair (Figure 9a–k). Long‐term microenvironmental imbalances are associated with TBI‐related complications.^[^ ^2a–e^ ^]^ Therefore, we also studied the role of Nano‐plumber in mitigating TBI‐related AD.^[^ ^27^ ^]^ The results showed that the nanoparticles significantly reduced Aβ accumulation in the injured brain region of mice, indicating their potential in reducing the incidence of TBI‐related AD (Figure 9l; Figure S21, Supporting Information).

The design of the Nano‐plumber incorporates a comprehensive focus on both safety and efficacy. In relation to safety, the process of gene delivery inherently induces cellular toxicity, and the prolonged overexpression of VEGF‐C may potentially trigger immune activation reactions unrelated to the therapeutic purpose. To address these concerns, Nano‐plumber employs a calcium phosphate‐compressed plasmid encoding VEGF‐C, enabling excellent transient transfection capability that allows for safe and controllable overexpression of the cytokine within a specific timeframe. To further enhance safety, Nano‐plumber is modified with lactose, exhibiting specific affinity toward upregulated Gal‐3 receptors on the surface of activated microglia in the lesion environment. Consequently, this design minimizes potential impact on other vulnerable neuronal cells. In terms of effectiveness, Nano‐plumber additionally co‐delivers the small molecule drug pro‐DHA. This drug promptly exerts its effects in reversing the detrimental microenvironment and protecting blood vessels prior to the completion of VEGF‐C‐mediated reconstitution of meningeal lymphatics. By integrating transient and long‐term regulatory mechanisms, Nano‐plumber operates as an exceptionally efficient dual‐delivery system for both chemical and gene‐based therapeutics, covering the entire course of TBI.

The importance of meningeal lymphatic vessels in TBI has been largely overlooked in the majority of studies. In fact, long‐term impairment of meningeal lymphatic vessel function after TBI is a significant factor contributing to the inability to effectively clear harmful substances from the brain microenvironment during the chronic phase.^[^ ^7^, ^9^, ^10^, ^28^ ^]^ Thus, enhancing the drainage capacity of meningeal lymphatic vessels represents a novel approach to achieving sustained and autonomous regulation of the brain microenvironment. Currently, the primary intervention for meningeal lymphatic vessels involves intracranial injection of lentivirus to transfuse VEGF‐C.^[^ ^10^, ^28^ ^]^ However, our developed nanocarrier system for intravenous injection may be more clinically applicable. Notably, the mechanisms underlying the impairment of meningeal lymphatic drainage function after TBI are not fully understood, and future treatments aimed at preventing this impairment may have a positive impact on long‐term prognosis of the disease.^[^ ^29^ ^]^ Additionally, the mechanism by which cerebrospinal fluid (CSF) enters and exits meningeal lymphatic vessels is still a topic of considerable debate. Further investigations are needed to elucidate the types of large molecules and cells that can be transported through the meningeal lymphatic system under various disease states.^[^ ^30^ ^]^

Considering the glymphatic‐lymphatic system as a cohesive entity, the regulation of blood vessels and lymphatic vessels is interdependent, as both play vital roles in maintaining the self‐regulating environmental homeostasis of brain tissue.^[^ ^7^, ^9^, ^10^, ^31^ ^]^ In this study, we examine the role of blood vessels in regulating the TBI microenvironment from a novel perspective. On one hand, severe damages to blood vessels after TBI exacerbate microenvironmental disruption, while our nano‐drug delivery system incorporating pro‐DHA has been shown to protect endothelial cells, thus improving the microenvironment (Figure 5a–j). On the other hand, the PVS is a critical component of the glymphatic‐lymphatic system, and normal vascular function can aid in the sustainable homeostatic maintenance of the meningeal lymphatic system (Figures 6 and 8).

Furthermore, efficient and rapid reversal of the acute‐phase microenvironment in TBI is also a crucial aspect. Previous knowledge of the epigenetic signals that control the function of microglia in vivo was limited. However, recent research has indicated that HDACs, particularly HDAC1 and HDAC2, play an essential role in a range of dysregulated processes in microglia, including inflammation activation and impaired phagocytosis.^[^ ^13^ ^]^ Thus, we have identified HDAC as a critical molecular target and employed pro‐DHA to achieve a more effective and comprehensive reshaping of microglial function (Figures 4a–e and 6a,c; Figure S19, Supporting Information).

In addition to TBI, other neurological disorders such as stroke, AD, epilepsy, and Parkinson's disease share similar pathological features.^[^ ^32^ ^]^ Patients with stroke, epilepsy, and Parkinson's disease may experience prolonged inflammatory states lasting months or even years. Prolonged inflammation exerts detrimental effects on patient recovery and neurofunctional restoration, encompassing disruptions in normal neuronal function, impediments to brain tissue repair and regeneration, and heightened risk of complications.^[^ ^32a,c,d^ ^]^ The therapeutic strategy of Nano‐plumber, involving rapid microenvironmental reversal and enhancement of the glymphatic‐lymphatic system function to maintain microenvironmental homeostasis, may offer a promising treatment approach for improving long‐term prognosis in patients with stroke, epilepsy, and Parkinson's disease. In the case of AD, the pathological hallmark resides in the excessive accumulation of amyloid plaques, playing a pivotal role as a pathogenic mediator of neurotoxicity and chronic inflammation.^[^ ^32b^ ^]^ Nano‐plumber, by enhancing the drainage function of the glymphatic‐lymphatic system, can assist in the clearance of harmful amyloid proteins, thereby preserving the dynamic homeostasis in the brain. Hence, this therapeutic approach holds potential applicability in the management of AD.

In order to facilitate clinical translation, several limitations of this study need to be addressed. First, although promising therapeutic effects were achieved in the CCI mouse model, these results need to be validated in clinical trials. Second, the pathological changes following the enhancement of meningeal lymphatic drainage with Nano‐plumber in TBI require further investigation. Finally, although we achieved a comprehensive and effective regulation of microglial through HDAC intervention, the exact mechanism of action remains to be fully elucidated. Moreover, the precise mechanisms underlying the vascular‐protective effects of pro‐DHA required further investigation.

In conclusion, given the distinctive pathological features and clinical treatment challenges of TBI, we have developed a novel nanostructure, Nano‐plumber, for the co‐delivery of pro‐DHA and VEGF‐C, enabling a sustainable and orderly regulation in injury microenvironment, and promoting long‐term neurological recovery after TBI. This platform technology reshapes the function of microglia and sustains perivascular drainage pathways, while promoting the drainage of meningeal lymphatic vessels. As a result, it effectively restores and maintains the homeostasis of the microenvironment after TBI, supporting neural regeneration and repair. Moreover, this approach holds promise for addressing a range of brain injury diseases with similar pathological features.

## 4 Experimental Section

### Ethical Regulations

All the animal experiments were performed in accordance with the guidelines evaluated and approved by Institutional Animal Care and Use Committee (IACUC), Fudan University School of Pharmacy (Ethical approval number: 2018‐03‐YJ‐CJ‐01).

### Materials

1,2‐dioleoyl‐sn‐glycero‐3‐phosphate (DOPA) was purchased from Avanti Polar Lipids (Alabaster, AL, USA). 1,2‐dimyristoyl‐sn‐glycero‐3‐phosphocholine (DMPC) was purchased from AVT Pharmaceutical Tech Co., Ltd (Shanghai, China). N‐(methoxy polyethylene glycol_2000_)−1,2‐distearoyl‐sn‐glycero‐3‐phosphoethanolamine (mPEG_2000_‐DSPE) and Gal‐PEG_2000_‐DSPE were obtained from Xi'an Ruixi biotech (Xi'an, China). mPEG‐PLA was kindly provided by East China University of Science and Technology. Octaarginine peptide (mc‐CR8C) was purchased from GL Biochem Ltd. (Shanghai, China). 1,1′‐dioctadecyl‐3,3,3′,3′‐tetramethylindotricarbocyanine iodide (DiR), 1,1′‐dioctadecyl‐3,3,3′,3′‐tetramethylindocarbocyanine perchlorate (DiI) and agar (70101ES76) were purchased from MeilunBio (Dalian, China). IGEPAL® CO‐520, Hoechst 33 258, 3‐[4,5‐dimethylthiazol‐2‐yl]−2,5‐diphenyl tetrazolium bromide (MTT), cou6 and docosahexaenoic acid (DHA) were purchased from Sigma‐Aldrich (St. Louis, MO, USA). Nitric oxide synthase assay kit, LPS, and enhanced mitochondrial membrane potential detection kits (JC‐1) were purchased from Beyotime (Shanghai, China). ELISA kits and ROS assay kit were provided by Multi Science (Hangzhou, China). Yeast extract (LP0021) and tryptone (LP0042) were obtained from OXOID (Hants, UK). Anti‐Iba1 primary antibody (ab289370), anti‐NeuN primary antibody (ab177487), anti‐CD31 primary antibody (ab281583), Anti‐Lyve1 primary antibody (ab218535), Anti‐Claudin‐5 primary antibody (ab131259), Anti‐ZO‐1 primary antibody (ab221547), goat anti‐rabbit IgG Alexa Fluor 488 secondary antibody (ab150077), goat anti‐rabbit IgG Alexa Fluor 594 secondary antibody (ab150080) and goat anti‐rabbit IgG Alexa Fluor 647 secondary antibody (ab150079) were purchased from Abcam (Cambridge, UK). Alexa Fluor 488 anti‐GFAP antibody (Cat No: 837 507) and anti‐β‐Amyloid, 1–42 primary antibody (Cat No: 805 509) were purchased from Biolegend (San Diego, USA). Anti‐mouse CD80‐FITC antibody and anti‐mouse CD206‐PE were purchased from eBioscience (San Diego, USA). All the other chemical reagents and solvents were purchased from Sinopharm Chemical Reagent Co., Ltd (Shanghai, China) unless specified.

### Cell Lines and Animals

HT22 cells were kindly provided by Prof. Gao Xiaoling of Shanghai Jiaotong University. bEnd.3 cells and BV2 cells were purchased from Chinese Academy of Science Cell Bank (Shanghai, China). PC‐12, bEnd.3, and BV2 cells were cultured in Dulbecco's modified Eagle's medium (DMEM, high glucose, Hyclone) containing 10% fetal bovine serum and 1% penicillin‐streptomycin solution. Male C57BL/6 mice (6–10 weeks old,18–22 g) were purchased from SLAC Animal Ltd. (Shanghai, China) and raised in a pathogen‐free facility with a 12 h light and dark cycle at 18–23 °C and 40–60% humidity and had free access to food and water. In terms of animal experiment studies, male mice were chosen. Male mice are less likely to die during the establishment of TBI models based on the historical experience.^[^ ^5b^ ^]^ This can reduce accidental death and help to ensure the objectivity of the studies.

### Synthesis and Characterization of pro‐DHA

The pro‐DHA was synthesized via an esterification reaction.^[^ ^14^ ^]^ Specifically, DHA (0.152 mmol) and dichloromethane (5 mL) were added to a 25 mL reaction flask and stirred until dissolved. Next, EDC (0.228 mmol), DMAP (0.138 mmol), and 2,6‐di‐tert‐butyl‐4‐methylphenol (BHT, 10 µL) in dichloromethane were added slowly in portions at room temperature under nitrogen protection, and the mixture was stirred for 2 hours. Subsequently, isopropanol (0.138 mmol) in dichloromethane (1 mL) was slowly added dropwise under nitrogen protection, and the mixture was refluxed for 12 h. TLC was employed to monitor the reaction progress, and once complete, the mixture was cooled to room temperature. Then the mixture was filtered, and the filtrate solvent was evaporated under reduced pressure. The resulting transparent colorless oil pro‐DHA was purified by column chromatography, and the yield was 53.8%. DHA, isopropanol, and pro‐DHA were dissolved in CDCl_3_ for 1H NMR spectroscopy comparison analysis, and pro‐DHA was dissolved in dichloromethane for ESI‐MS analysis.

### Preparation and Characterization of Gal‐pro‐DHA/pVEGFC@CaP

The inner cores of Gal‐pro‐DHA/pVEGFC@CaP were prepared using a water‐in‐oil microemulsions method as described previously with some modification.^[^ ^12c^ ^]^ Briefly, two separate microemulsions (20 mL each) consisting of cyclohexane/Igepal® CO‐520 (7:3, v/v) were prepared with constant stirring. A solution of pDNA (180 µg, 2.0 mg mL^−1^) was prepared and mixed with CaCl_2_ solution (300 µL, 2.5 m). To this solution, mc‐CR8C (25 µL, 8 mg mL^−1^) was added and then dropped into the microemulsion to form the calcium part. A solution of Na_2_HPO_4_ (300 µL, 12.5 mm) was also prepared and added to the other microemulsion. This microemulsion was mixed with the other microemulsion containing DNA/mc‐CR8C/CaCl_2_. After stirring the mixed microemulsion for 5 minutes, DOPA (300 µL, 20 mm) in chloroform was added, and the microemulsion was left to stir for another 45 min. An equal volume of 100% ethanol (40 mL) was added and the mixture was centrifuged at 12500×g for 20 min at 4 °C. The supernatant was discarded and the precipitates were washed twice with 100% ethanol to remove the cyclohexane and Igepal® CO‐520. The precipitates were then suspended in chloroform and store at −20 °C for further use.

To obtain the final Gal‐pro‐DHA/pVEGFC@CaP, cores (10 mg) and DMPC (5 mg) were mixed with mPEG2000‐DSPE (250 µL, 20 mm) and Gal‐PEG2000‐DSPE (50 µL, 20 mm) dissolved in chloroform, and then evaporated in vacuum to obtain a thin lipids film. 5% Glu solution was used to rehydrate the lipids film followed by sonication to obtain the final formulation. The DiI or DiR‐labeled CaP nanoparticles were prepared using the same procedure by adding 1% DiI or DiR to the lipids.

The particle size and zeta potential of Gal‐pro‐DHA/pVEGFC@CaP were measured using a Malvern ZetaSizer Nano series (Westborough, MA). TEM images of Gal‐pro‐DHA/pVEGFC@CaP were obtained using a TEM (TEM‐1400 Plus Electron Microscope, Leica, Germany). The LE and EE of pro‐DHA were determined by high‐performance liquid chromatography (HPLC) at a detector wavelength of 214 nm. The LE and EE of plasmid were determined according to the previous report.^[^ ^33^ ^]^ The cores were collected and lysed using pH 4.0 acetic acid buffer, and the peptide/DNA complex was dissociated in protease K solution at 37 °C for 1 h. Hoechst 33 258 nucleic acid stain was added and measured by fluorescence spectrometry. The DNA encapsulation efficiency was calculated by using a standard curve obtained by mixing blank cores with known concentrations of peptide/pDNA complexes.

### Stability of Gal‐Pro‐DHA/pVEGFC@CaP

The long‐term storage stability of Gal‐pro‐DHA/pVEGFC@CaP was assessed over a period of three months at 4±2 °C. The dilution stability of Gal‐pro‐DHA/pVEGFC@CaP was evaluated in both PBS and 10% FBS over a two‐week period. Specifically, 0.1 mL Gal‐pro‐DHA/pVEGFC@CaP suspension was diluted in 4.9 mL PBS and 10% FBS, respectively, and then stored at 4±2 °C. The particle size was measured by DLS analysis.

### The Determination of Plasmid Concentration

Hoechst 33 258 stock (1 mg mL^−1^ in distilled H_2_O) was previously prepared and sterilized by filtration through a 0.22 µm filter and stored at 4 °C in a light tight container. Working assay solution was prepared by adding 1 µL of stock solution for every 1 mL of assay buffer. Nano‐plumber was dissolved in lysis buffer (2 mmol EDTA and 0.05% Triton X‐100 in pH 7.8 Tris buffer) at 65 °C for 10 min. Then, working assay solution was added, and samples were assessed via fluorescence spectrometry with a 360‐nm, 20‐nm bandwidth, excitation filter, and a 460‐nm, 20‐nm bandwidth emission filter. Standard curves were generated using blank CaP along with known concentrations of plasmid.

### Preparation of Cou6‐Labeled mPEG‐PLA Nanoparticles

To evaluate the phagocytic capacity of BV2, coumarin 6 (cou6)‐labeled mPEG‐PLA nanoparticles were used as the fluorescent marker. Cou6‐labeled mPEG‐PLA nanoparticles were prepared by emulsion/solvent evaporation as described previously.^[^ ^34^ ^]^ Briefly, cou6 (0.1 mg) and mPEG‐PLA (10 mg) were dissolved in dichloromethane (DCM, 1.0 mL). Then, 1% sodium cholate solution (2.0 mL) was slowly added to the surface of the DCM solution, and the mixture was subjected to ultrasonication under an ice bath. The obtained emulsion was dispersed in 0.5% sodium cholate solution (8.0 mL). After removing DCM by evaporation, the nanoparticle suspension was centrifuged at 20 000×g for 1.0 h and resuspended in 5% Glu solution. Finally, a 1.5 × 20 cm sepharose CL‐4B column was used to remove the free cou6.

### Investigation of Phagocytic Capacity on BV2 Cells

BV2 cells were seeded at a density of 5000 cells per well in 96‐well plates and cultured for 24 h for attachment. Subsequently, the culture medium was replaced with DMEM containing different concentrations of free pro‐DHA or Vorinostat(commercially available agents). After incubation for 24 h, the medium was discarded, and cou6‐labeled mPEG‐PLA nanoparticles (at the cou6 concentration of 50 ng mL^−1^) dispersed in DMEM were added and incubated for an additional hour at 37 °C. After that, BV2 cells were washed with PBS, fixed with 4% paraformaldehyde, and stained with 2 µg mL^−1^ Hoechst 33 258. The cells were analyzed using a KineticScan® HCS Reader (Version 3.1, Cellomics Inc., Pittsburgh, PA, USA).

### Cytotoxicity Assay

The cytotoxicity of pro‐DHA in BV2 cells was evaluated using the MTT assay. Briefly, BV2 cells were seeded at a density of 5000 cells per well in 96‐well plates and cultured for 24 h for attachment. The culture medium was then replaced with DMEM containing varying concentrations of free pro‐DHA, with free DMEM without pro‐DHA serving as the control group. After incubation for 24 h, MTT solution (5 mg mL^−1^) was added and incubated with cells for an additional 4 h at 37 °C. Formazan crystals produced by viable cells were dissolved in 200 µL of DMSO, and the absorbance was measured at 490 nm using a microplate reader (Thermos Multiskan MK3, USA).

### In Vitro Cell Uptake Study

Cellular uptake was conducted using confocal microscopy. BV2 cells were either stimulated with 100 ng/mL LPS or left unstimulated and cultured in confocal dishes until 80%−90% confluent. DiI‐labeled Gal‐pro‐DHA/pVEGFC@CaP was added to each dish and incubated for 1 and 2 h. The cells were subsequently washed with PBS and fixed with 4% paraformaldehyde. Hoechst 33 258 staining was performed at a concentration of 2 µg mL^−1^ for 10 min. Confocal laser scanning microscopy (CLSM, LSM710, Leica, Germany) was utilized to visualize the cells.

Quantitative analysis of cell uptake was conducted using flow cytometry. BV2 cells were pre‐stimulated with 100 ng mL^−1^ LPS or left unstimulated and cultured in a 6‐well plate until 80%−90% confluent. DiI‐labeled Gal‐pro‐DHA/pVEGFC@CaP was added to each well and incubated for 1 and 2 h. The cells were then washed with PBS, resuspended in PBS, and analyzed using a flow cytometer (Beckman, Coulter).

The cell uptake inhibition experiment was analyzed using flow cytometry. BV2 cells were pre‐stimulated with 100 ng mL^−1^ LPS or left unstimulated and cultured in 6‐well plates until 80%−90% confluence was achieved. The medium was subsequently replaced with medium containing 100 µg mL^−1^ lactose, and the cells were incubated for 15 min before being switched back to regular medium. DiI‐labeled Gal‐pro‐DHA/pVEGFC@CaP was added to each well and the cells were incubated for 1 or 2 h. The cells were then washed with PBS, resuspended in PBS, and measured using a flow cytometer (Beckman, Coulter).

### In Vitro Transfection Assay

To evaluate the transfection efficiency, flow cytometry and confocal assays were employed. BV2 cells were pre‐stimulated with 100 ng mL^−1^ LPS or left unstimulated and cultured until 80–90% confluent in 6‐well plates. EGFP‐Gal‐pro‐DHA/pVEGFC@CaP, loaded with 1 or 2 µg of plasmid, was added to the cells in the presence of Opti‐MEM. The negative control consisted of nanoparticle (Gal‐pro‐DHA/pVector@CaP) loaded with non‐EGFP coding vector plasmid. Then the medium was replaced with DMEM 6 h after the transfection and incubated for another 24 h. After the imaging, the expression of EGFP was measured via flow cytometry and observed by confocal laser scanning microscopy (CLSM, LSM710, Leica, Germany). The experiments were repeated three times independently.

### Mitochondrial Membrane Potential Assay

To assay the mitochondrial membrane potential, confocal assays were employed. In brief, BV2 cells were pre‐stimulated with 100 ng mL^−1^ LPS or left unstimulated and cultured until 80–90% confluent in confocal culture dish. The culture medium was removed, and cells were washed once with PBS. Then, 1 mL cell culture medium and 1 mL JC‐1 staining working solution were added and mixed thoroughly. The cells were incubated at 37 °C in a cell culture incubator for 20 min. After incubation, the supernatant was removed, and cells were washed twice with JC‐1 staining buffer. Next, 2 mL of cell culture medium was added, and the cells were observed under a laser confocal microscope. ImageJ was used to evaluate the fluorescence intensity on each image to determine the level of the mitochondrial membrane potential.

### In Vitro Microglial Polarization Assay

To investigate the macrophage polarization in vitro, BV2 cells were planted in 6‐well plates and stimulated with 100 ng mL^−1^ LPS for 24 h, followed by treatment with 5% Glu (control group), free pro‐DHA, Gal‐pro‐DHA@CaP, Gal‐pVEGFC@CaP, PEG‐pro‐DHA/pVEGFC@CaP and Gal‐pro‐DHA/pVEGFC@CaP(an equivalent pro‐DHA amount of 10 µm) for 24 h. Subsequently, the cells were collected and washed with PBS. Anti‐mouse CD80 and CD206 antibody were added and incubated for 45 min at 4 °C, respectively. Then the cells were washed with PBS and detected by flow cytometry (Beckman, coulter).

### Detection of iNOS in BV2

To detect the iNOS level in vitro, BV2 cells were planted in 96‐well plates, stimulated with 100 ng mL^−1^ LPS for 24 h, followed by adding 5% Glu (control group), Free pro‐DHA, Gal‐pro‐DHA@CaP, Gal‐pVEGFC@CaP, PEG‐pro‐DHA/pVEGFC@CaP and Gal‐pro‐DHA/pVEGFC@CaP (an equivalent pro‐DHA amount of 10 µm) and incubated for 24 h. The culture medium was then removed, and 100 µL of NOS detection buffer was added to each well, followed by the addition of 100 µL of detection reaction solution and gentle mixing. The plate was then incubated at 37 °C for 20–60 min in a cell culture incubator. The fluorescence intensity was measured directly using a fluorescence microplate reader with excitation and emission wavelengths set to 495 nm and 515 nm, respectively. Wells without cells served as blank controls.

### Detection of ROS in Different Cells

To detect the ROS level in different cells, cells were planted in 96‐well plates and stimulated with 100 ng mL^−1^ LPS for 24 h, followed by treatment with 5% Glu (control group), free pro‐DHA, Gal‐pro‐DHA@CaP, Gal‐pVEGFC@CaP, PEG‐pro‐DHA/pVEGFC@CaP and Gal‐pro‐DHA/pVEGFC@CaP(an equivalent pro‐DHA amount of 10 µm) for 24 h. The cells were washed with PBS and incubated with 10 µm DCFH‐DA for 1 h at 37 °C. After the incubation, the medium was removed and the cells were washed with PBS three times. The fluorescence intensity was measured by a Microplate Reader (Synergy H1, BioTek) at excitation and emission wavelengths at 488 and 525 nm respectively.

### Wound Healing Assay

The migration of bEnd.3 cells was evaluated by the wound healing assay. Briefly, bEnd.3 cells were seeded in a 24‐well plate and cultured for 24 h until the bottom monolayer cells spread to more than 90% of the plate. Then the cell monolayer was scratched using a sterilized 1 mL pipette tip. Next, Transwell inserts (with 0.8 µm pore diameter, Corning, USA) were placed on the wells. BV2‐M0, BV2‐M1, and Gal‐pro‐DHA/pVEGFC@CaP‐treated BV2‐M1 cells were inoculated in the upper chamber. After incubation for 0, 12, 24, and 36 h, the scratch area was recorded by an inverted microscope. ImageJ was used to evaluate the size of the denuded area on each image to quantify the extent of wound healing.^[^ ^35^ ^]^

### Controlled Cortical Impact (CCI) Mouse Model

The CCI models were established as previously reported with some modification.^[^ ^5^ ^]^ In brief, the animals were anesthetized intraperitoneally anesthetized with sodium pentobarbital. The bregma and the right parietal bone of the mice were exposed, and a 4 mm‐diameter burr hole in the skull was bored on the right side of the midline and 1 mm posterior to bregma. The bone flap was removed to entirely expose the dura mater. The injury model was generated by striking the dura with an impactor tip of 3 mm in diameter. After the impact, the wound was immediately sutured. Then the mice were placed in an animal intensive care unit until palinesthesia at the temperature of 37 °C. Sham‐operated mice were treated underwent the same way but without the impact procedure.

### NIRF Imaging

The CCI mice were divided into two groups (*n* = 3) and intravenously injected with DiR, DiR‐PEG‐pro‐DHA/pVEGFC@CaP, and DiR‐Gal‐pro‐DHA/pVEGFC@CaP. At 1 h, 2 h, 4 h, 8 h, 12 h, 24 h, 48 h, and 72 h after injection, the CCI mice were anesthetized with isoflurane and imaged using an IVIS imaging system to detect the distribution of the formulations in the body. At the last time point, the mice were euthanized, and the heart, liver, spleen, lung, kidney, and brain tissues were harvested. The fluorescence distribution data of the excised organs were collected using an IVIS imaging system, and the DiR intensity values of each organ were semi‐quantitatively analyzed using analysis software.

### Tissue Processing

Following transcardial perfusion with saline and 4% paraformaldehyde for 15 min, the skullcap was harvested and fixed in 2% paraformaldehyde overnight. The meninges were subsequently removed from the skullcap. Brains were collected and fixed with 4% paraformaldehyde for 48 h at 4 °C. Then the brains were gradually dehydrated with 15 and 30% sucrose solution. For immunofluorescence staining, brains were embedded in OCT (Sakura, Torrance, CA, USA), and 10‐µm‐thick sections were sliced by a cryostat (Leica, CM3050S).

### Flow Cytometry Assay

To analyze in vitro polarization of microglia and in vitro transfection of microglia, BV2 cells treated with the formulation were collected, washed with PBS, and stained with fluorescently labeled antibodies. After labeling, the cells were washed with PBS, fixed with 4% paraformaldehyde, and subjected to flow cytometry analysis (Beckman,coulter). The acquired data were analyzed using FlowJo software (version X10, Tree Star, USA).

### Immunohistochemistry (IF) Chemistry Analysis

IF staining was performed on frozen sections of injury brains. The frozen sections were permeabilized and blocking in Immunol Staining Blocking Buffer (Beyotime Biotechnology Co., Ltd, Nantong, China) at room temperature for 1 h. Then the sections were incubated with fluorescently labeled antibodies or primary antibodies followed by secondary Alexa Flour 488, Alexa Flour 594, or Alexa Flour 647‐labeled goat anti‐rabbit IgG antibodies. Finally, the sections were stained with Hoechst 33 258 and observed using a fluorescence microscope (Leica DMI 4000B, Germany). The analysis of positive signals in images was performed by Image J software.

### In Vitro OGD/R Model

2 × 10^4^cells per pore bEnd.3 were seeded onto a 96‐well plate and placed in an incubator at 37 °C, 5% CO_2_ for 24 h. The ordinary DMEM medium was then replaced with glucose‐free DMEM medium and the cells were transferred to a three‐gas incubator with 92% N_2_, 3% O_2_, and 5% CO_2_ for 10 h, followed by reoxygenation in normal culture conditions with ordinary DMEM medium for 2 h at 37 °C in 5% CO_2_.^[^ ^36^ ^]^

### ELISA Assay

To investigate the changes in the microenvironment during the acute and chronic phases following TBI, an ELISA assay was performed to quantify pro‐inflammatory cytokines IL‐1β, IL‐6, TNF‐α, and iNOS, as well as anti‐inflammatory cytokines IL‐4, IL‐10, TGF‐β, and Arg‐1 in injured brain tissue at 7 and 28 days post‐injury. All specimens were processed and determined according to the manufacturer's protocol.

### Measurement of Neurological Scores

Neurological scores were assessed 72 h after administration using a 6‐point scale. 0, normal and active condition; 1, could not extend right forepaw completely; 2, circle to the right side; 3, could not stand up and fell to the right side; 4, no spontaneous movement; 5, no response to stimulation or death.^[^ ^37^ ^]^

### Behavior Tests

For behavior tests, mice were trained for 5 days before tests. Adhesive tests were conducted three times daily, while the cylinder test and forelimb placing test were conducted once daily. All tests were carried out on day 28 post‐CCI.

For rotarod test, Rotor‐Rod motor function system (San Diego Instruments, San Diego, CA, USA) was employed. Mice were placed onto rotating cylinders, which accelerated linearly from 0 to 50 rpm over a 5‐minute period. The latency to fall was recorded as the average of 5 separate runs for each animal.^[^ ^5c^ ^]^

For adhesive test, a 10×10 mm^2^ sticker was gently applied to the paralyzed forepaw using forceps. Next, the mouse was placed back in its home cage. The time duration from the start to the time the mouse starts to contact the sticker was recorded as time to touch. The duration from the start to the time the mouse successfully tears down the sticker was recorded as time to remove.

For cylinder test, mice were placed into a transparent cylinder (height: 35 cm; diameter: 15 cm). The whole process was videotaped by a camera for 5 min from above. The number of contacts by forepaws (left, L; right, R; both, B) was measured, and the asymmetric rate was calculated as (L − R)/(L + R +B) × 100 (%).^[^ ^38^ ^]^

For forelimb placing test, hold the back of the mouse so that the limbs are suspended, touch the beard to the edge of the table corner, and test the activity of the forelimb on the ipsilateral side. This action has different degrees of damage when the brain is injured. Mice were tested ten times, and the score was calculated as the percentage of the number of times the forelimbs touched the corner edge of the table.

### Morris Water Maze Assay

The water maze was filled with white dyed water and a platform fixed 3 cm beneath the water surface. Mice were trained to arrive at the platform from random starting positions around the maze border. Each quadrant was tested randomly. The learning trials were conducted four times a day, with 30‐minute intervals between each trial, for 7 consecutive days prior to the assay. During training, if the experimental mouse succeeded to find and stand on the platform within 120 s, it would be allowed to stay on the platform for 20 s. If it failed to find the platform, the tester would pick it up and place it on the platform for 20 s. To assess the neurological function, each trained mouse was allowed to swim freely for 120 s from a starting location far away from the platform 28 days after the CCI model establishment. The paths, latency, and swimming distance were recorded using a computerized video tracking system.^[^ ^39^ ^]^

### Statistical Analysis

All data were expressed as mean ± SD and analyzed using Prism 9.0.0. The statistical analysis was performed using one‐way ANOVA for comparison of multiple groups.

## Conflict of Interest

The authors declare no conflict of interest.

## Supporting information

Supporting Information

## Data Availability

The data that support the findings of this study are available from the corresponding author upon reasonable request.
