# Supplementary material for: Nano‐Plumber Reshapes Glymphatic‐Lymphatic System to Sustain Microenvironment Homeostasis and Improve Long‐Term Prognosis after Traumatic Brain Injury
Source: Adv Sci (Weinh). 2023 Oct 22;10(34):2304284. doi: 10.1002/advs.202304284 (PMC10700187; doi:10.1002/advs.202304284)
Supplement: Supplementary file 1 — Supporting Information [file ADVS-10-2304284-s001.pdf]

ADVANCED SCIENCE
Open Access

Supporting Information

for *Adv. Sci.*, DOI 10.1002/advs.202304284

Nano-Plumber Reshapes Glymphatic-Lymphatic System to Sustain Microenvironment Homeostasis and Improve Long-Term Prognosis after Traumatic Brain Injury

Shiqiang Tong, Laozhi Xie, Xiaoying Xie, Jianpei Xu, Yang You, Yinzhe Sun, Songlei Zhou, Chuchu Ma, Gan Jiang, Fenfen Ma, Zhihua Wang, Xiaoling Gao and Jun Chen**

Supporting Information

Nano-plumber Reshapes Glymphatic-lymphatic System to Sustain Microenvironment Homeostasis and Improve Long-term Prognosis after Traumatic Brain Injury

Shiqiang Tong, Laozhi Xie, Xiaoying Xie, Jianpei Xu, Yang You, Yinzhe Sun, Songlei Zhou, Chuchu Ma, Gan Jiang, Fenfen Ma, Zhihua Wang, Xiaoling Gao, and Jun Chen**

S. Tong, L. Xie, X. Xie, J. Xu, Y. You, Y. Sun, S. Zhou, C. Ma, F. Ma, J. Chen
Department of Pharmaceutics, School of Pharmacy & Shanghai Pudong Hospital, Fudan University, Shanghai, 201203, China.
Key Laboratory of Smart Drug Delivery, Ministry of Education, School of Pharmacy, Fudan University, Shanghai, 201203, China.
E-mail: chenjun@fudan.edu.cn

G. Jiang, X. Gao
Department of Pharmacology and Chemical Biology, State Key Laboratory of Oncogenes and Related Genes, Shanghai Universities Collaborative Innovation Center for Translational Medicine, Shanghai Jiao Tong University School of Medicine, Shanghai, 200025, China.
E-mail: shellygao1@sjtu.edu.cn

F. Ma
Department of Pharmacy, Shanghai Pudong Hospital, Fudan University, Shanghai, 201399, China.

Z. Wang
Department of Emergency, Shanghai Pudong Hospital, Fudan University Pudong Medical Center, Shanghai, 201399, China.

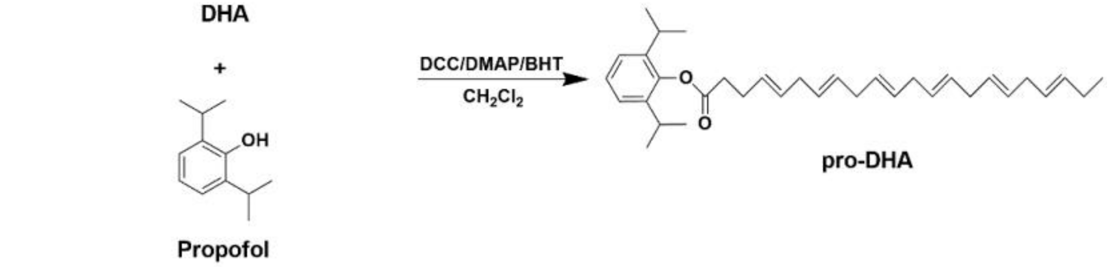

Figure S1. Synthetic route of pro-DHA.

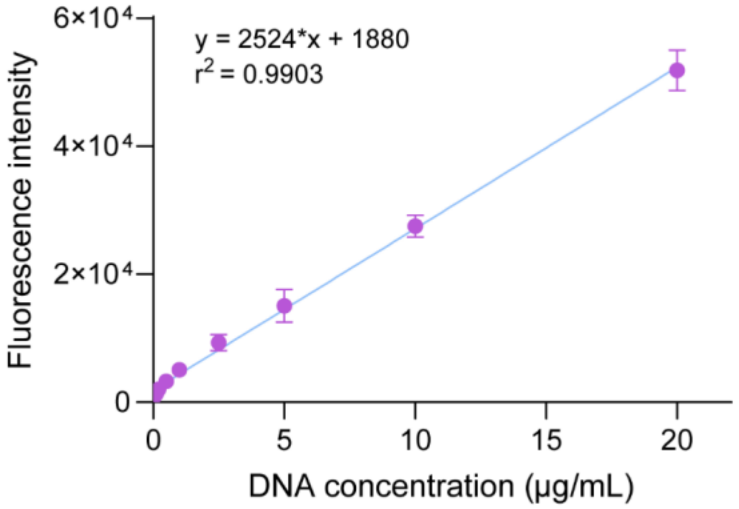

Figure S2. Concentration curve from 0.0 to 20 μg/mL with linear regression analysis (n = 5). Data represent mean ± SD of three independent replicates.

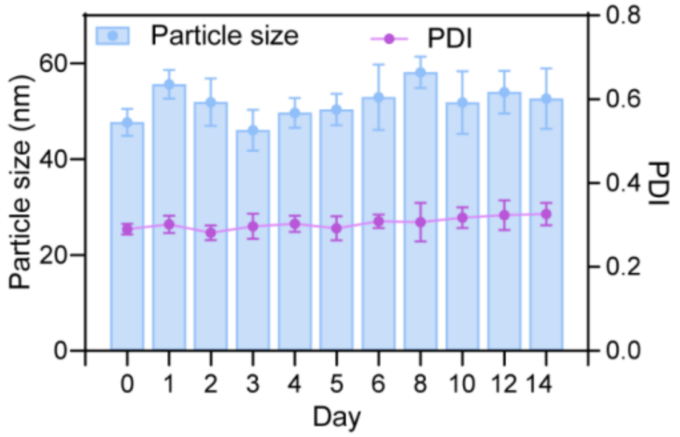

Figure S3. Long-term stability of Nano-plumber in 10% FBS media (n = 3). Data were expressed as mean ± SD.

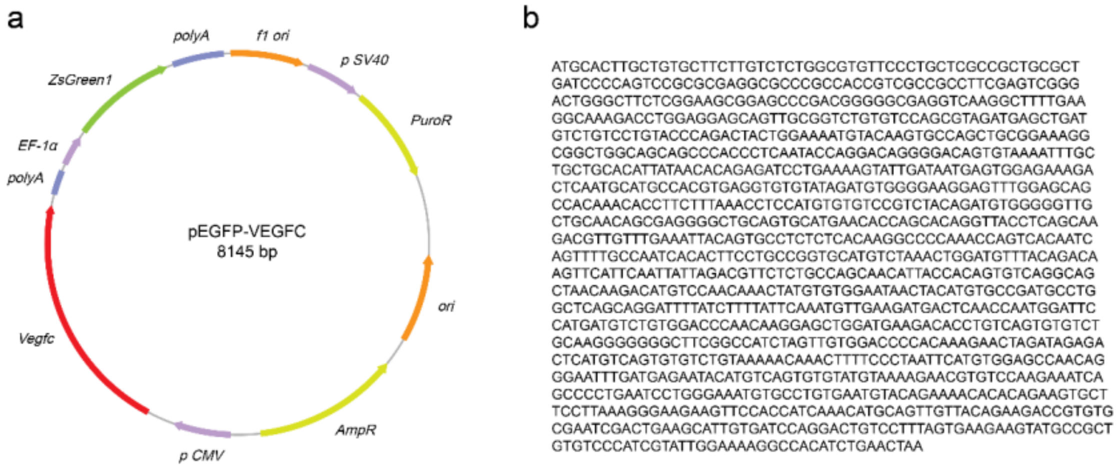

Figure S4. Representative scheme and sequence of plasmid. a) Representative scheme of plasmid simutaneously encoding VEGF-C and EGFP. b) The sequence encoding VEGF-C in the plasmid.

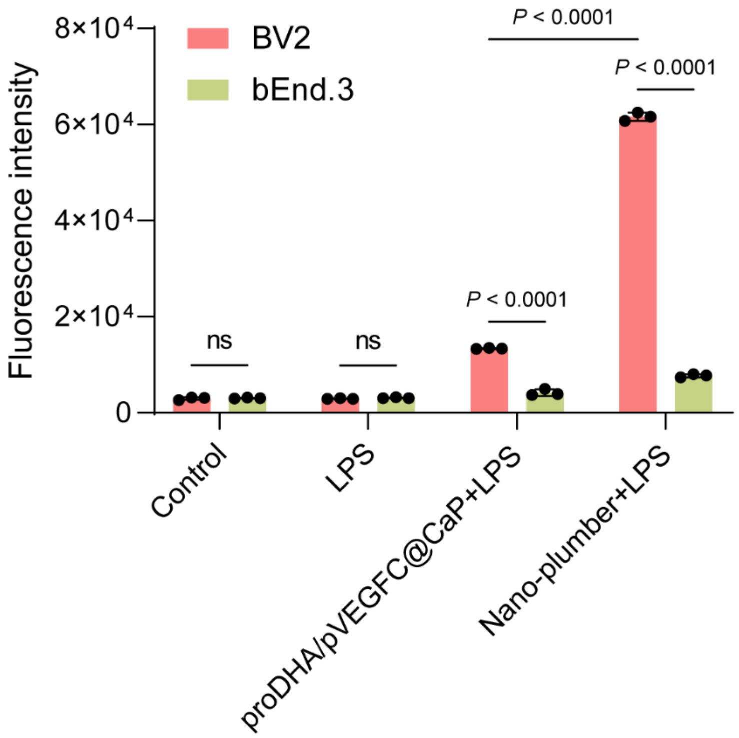

Figure S5. Cell-selective uptake of Nano-plumber ($n = 3$). Data represent mean ± SD of three independent biological replicates. Statistical analyses are performed using one-way ANOVA with Tukey's post hoc test.

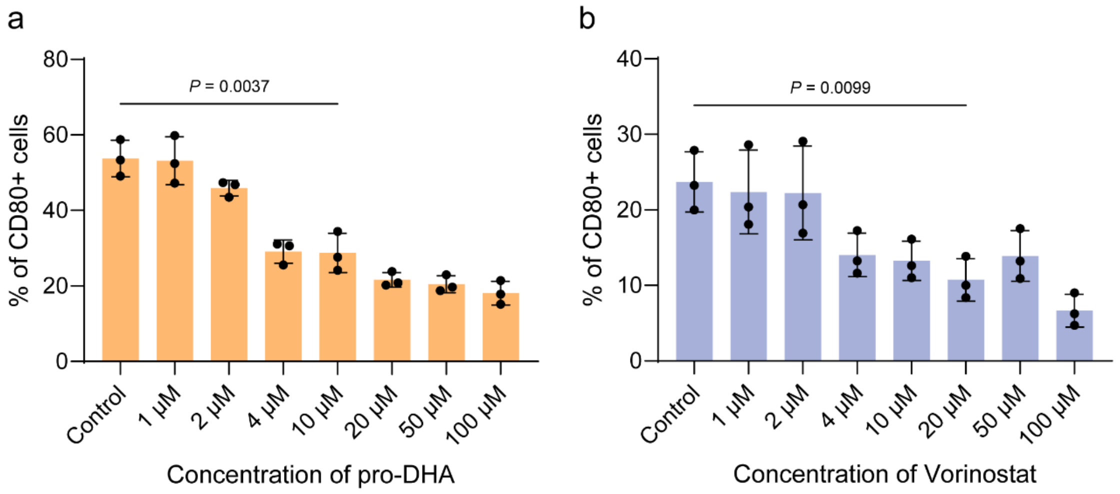

Figure S6. The anti-inflammatory effects of pro-DHA and Vorinostat on microglial. a, b) Flow cytometry analysis of $CD80^{+}$ cells in activated BV2 cells with different concentrations of (a) pro-DHA or (b) Vorinostat treatments ($n = 3$). Data represent mean ± SD of three independent biological replicates. Statistical analyses are performed using one-way ANOVA with Tukey's post hoc test.

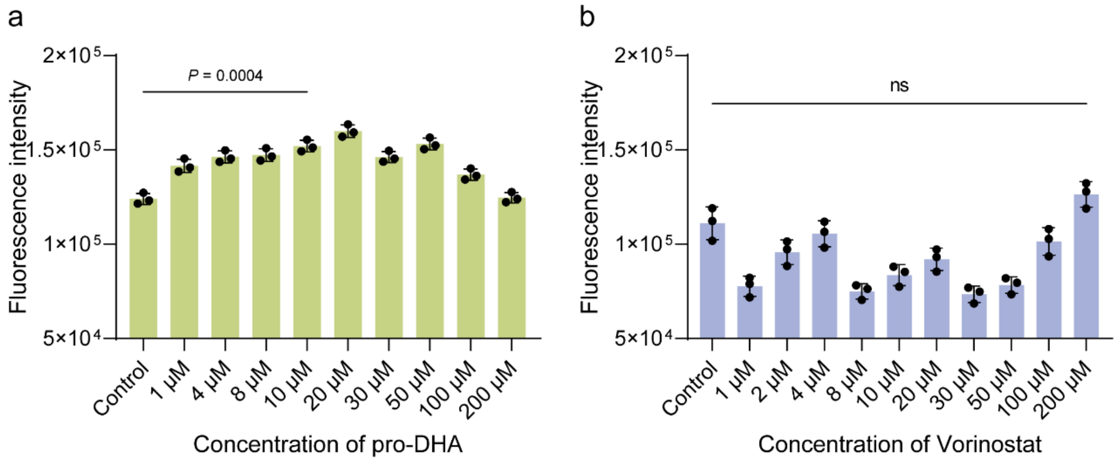

Figure S7. The modulatory effects of pro-DHA and Vorinostat on microglial phagocytosis capacity. a, b) Flow cytometry analysis of the phagocytic ability of dysfunctional BV2 cells with different concentrations of (a) pro-DHA or (b) Vorinostat treatments ($n = 3$). Data represent mean ± SD of three independent biological replicates. Statistical analyses are performed using one-way ANOVA with Tukey's post hoc test.

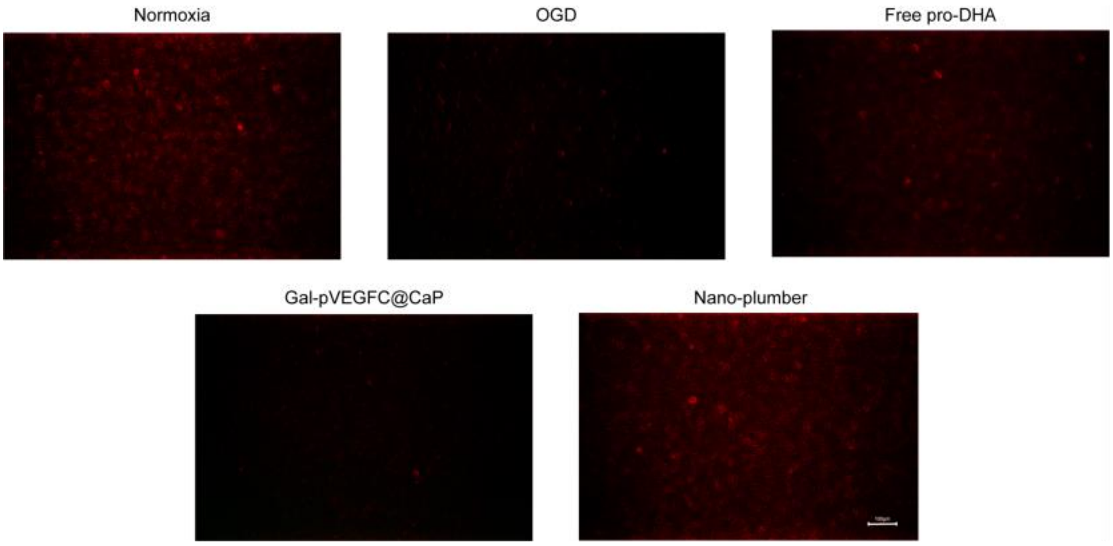

Figure S8. Representative images of the expression of Claudin-5 on bEnd.3 cells with different treatments after OGD (red: claudin-5). The experiments were repeated three times independently.

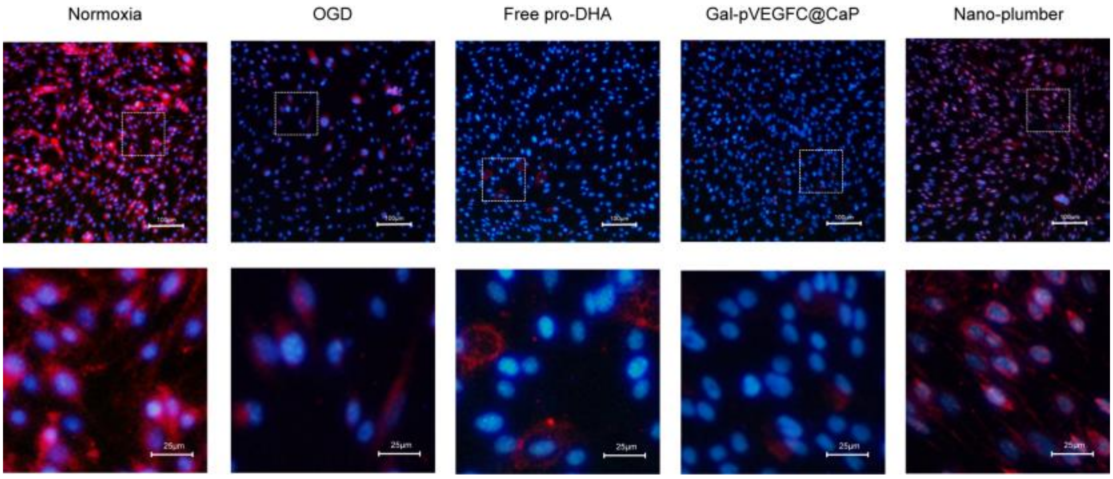

Figure S9. Representative images of the expression of ZO-1 on bEnd.3 cells with different treatments after OGD (blue: Hoechst, red: ZO-1). The experiments were repeated three times independently.

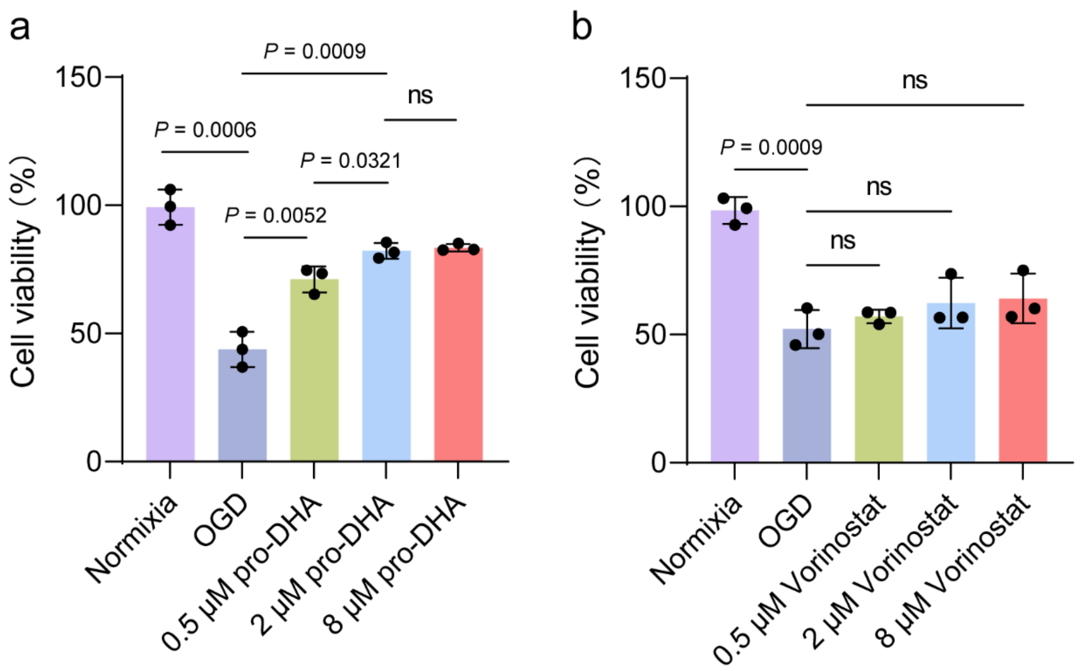

Figure S10. The protective effects of pro-DHA and Vorinostat on bEnd.3 cells. a, b) Cell viability of the bEnd.3 cells with different concentrations of (a) pro-DHA or (b) Vorinostat treatments after OGD (n = 3). Data represent mean ± SD of three independent biological replicates. Statistical analyses are performed using one-way ANOVA with Tukey's post hoc test.

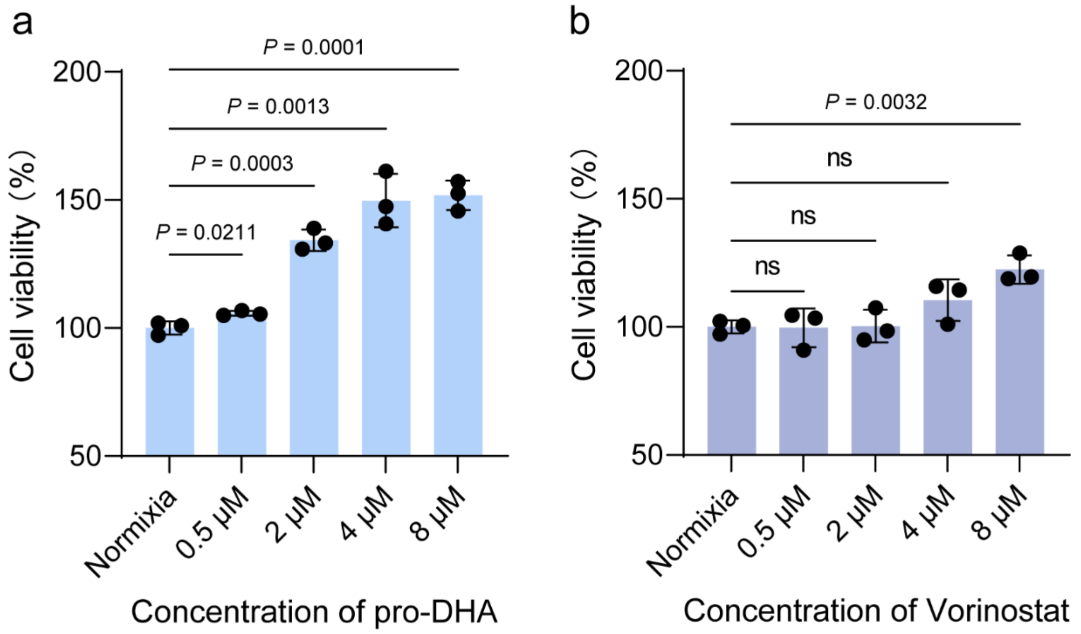

Figure S11. The proliferative effects of pro-DHA and Vorinostat on bEnd.3 cells. a, b) Cell proliferation of the bEnd.3 cells with different concentrations of (a) pro-DHA or

(b) Vorinostat treatments (n = 3). Data represent mean ± SD of three independent biological replicates. Statistical analyses are performed using one-way ANOVA with Tukey's post hoc test.

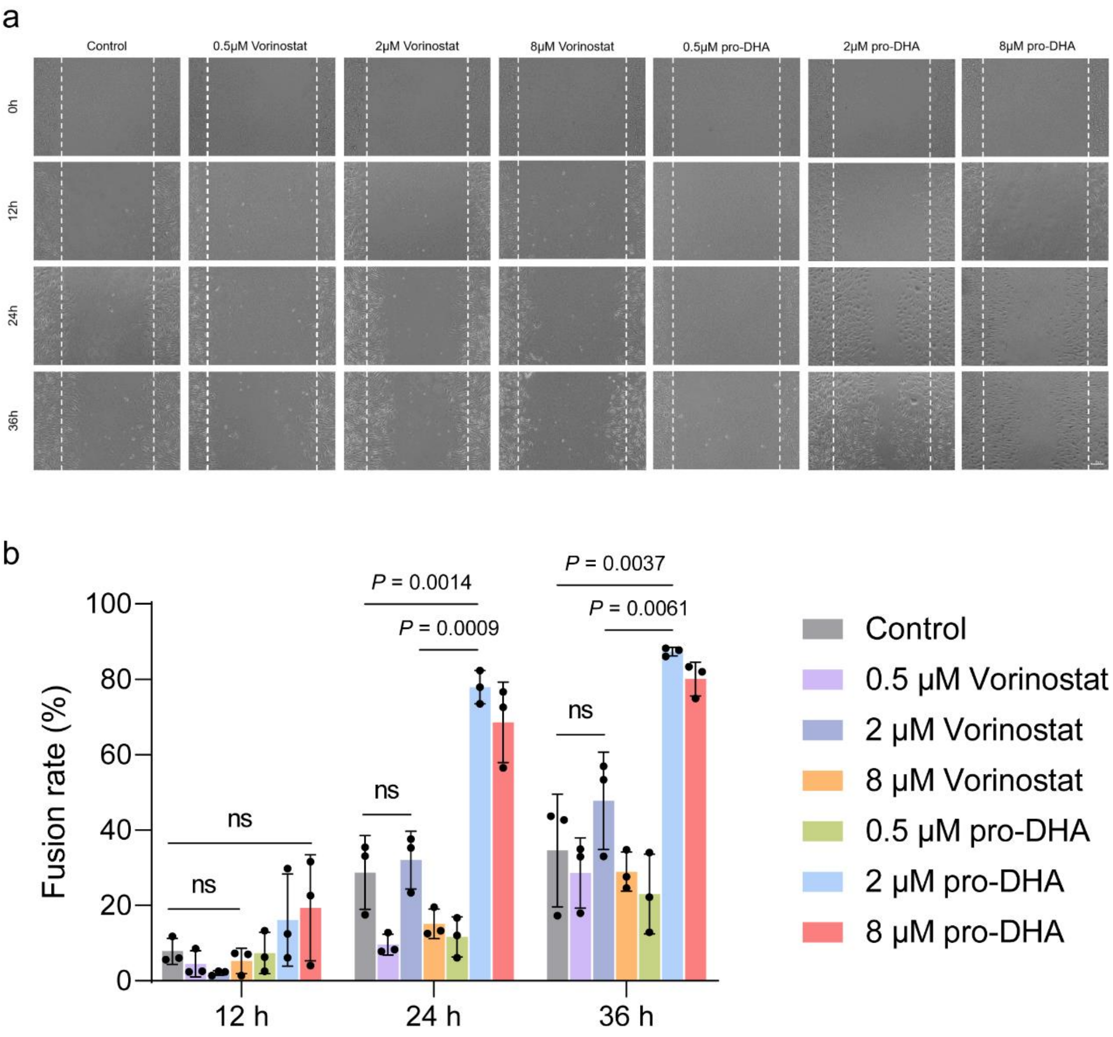

Figure S12. The migratory effects of pro-DHA and Vorinostat on bEnd.3 cells. a) Brightfield images of the wound healing process of bEnd.3 in basolateral well after different concentrations of pro-DHA or Vorinostat treatments. b) Quantification analysis of bEnd.3 fusion rate (n = 3). Data represent mean ± SD of three independent biological replicates. Statistical analyses are performed using one-way ANOVA with Tukey's post hoc test.

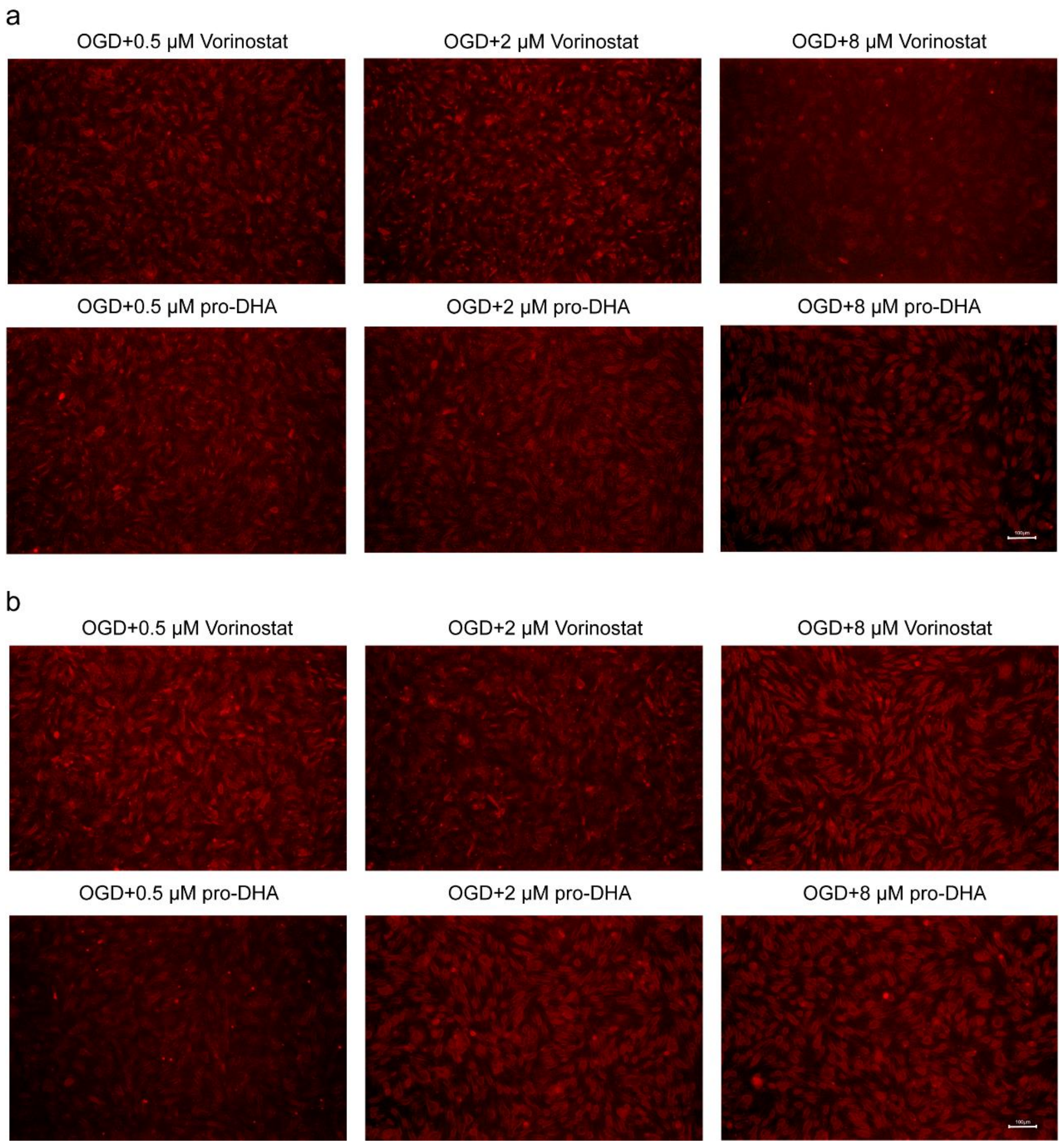

Figure S13. The promotion of tight junction formation in bEnd.3 cells by pro-DHA and Vorinostat. a, b) Representative images of the expression of (a) Claudin-5 and (b) ZO-1 on bEnd.3 cells with different concentrations of pro-DHA or Vorinostat treatments after OGD. The experiments were repeated three times independently.

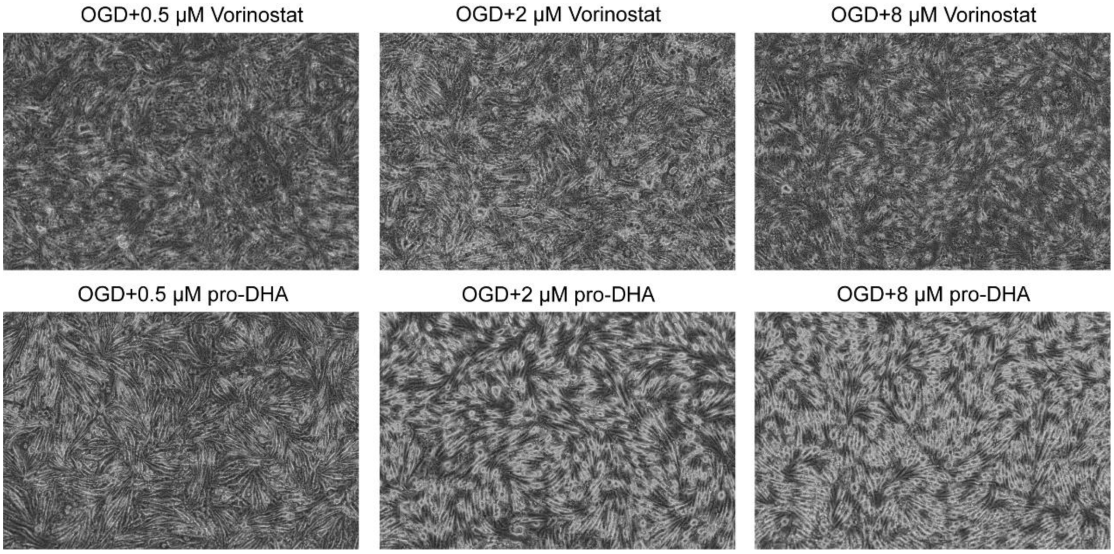

Figure S14. Cell morphology of bEnd.3 cells with different concentrations of pro-DHA or Vorinostat treatments after OGD. The experiments were repeated three times independently.

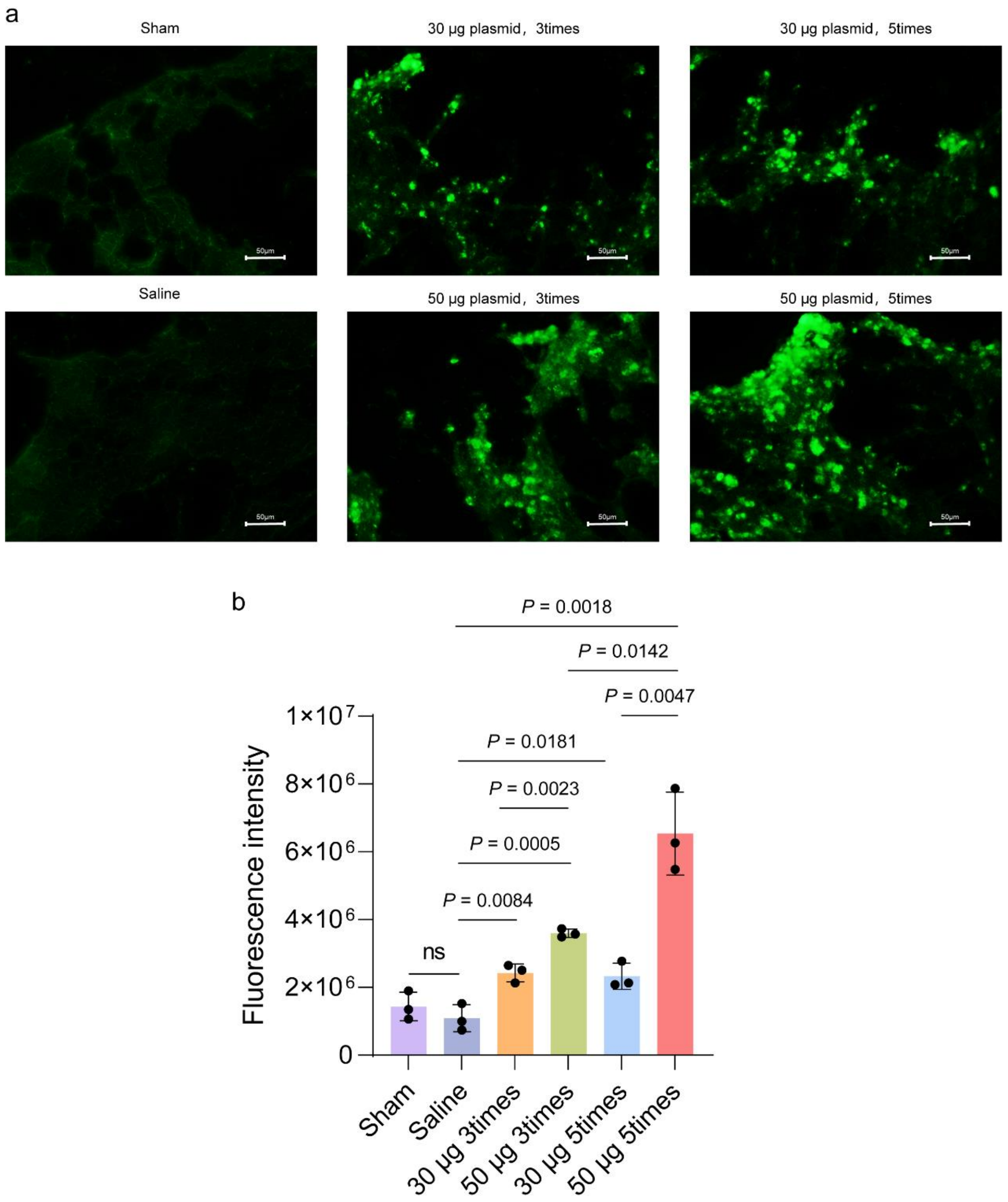

Figure S15. The transfection efficacy of Nano-plumber using different administration protocols *in vivo*. a) Representative images of transfection of Nano-plumber using different administration protocols *in vivo*. b) Quantitative analysis showing the fluorescence intensity of GFP corresponding to (a) (n = 3). Data represent mean ± SD of three independent biological replicates. Statistical analyses are performed using one-way ANOVA with Tukey's post hoc test.

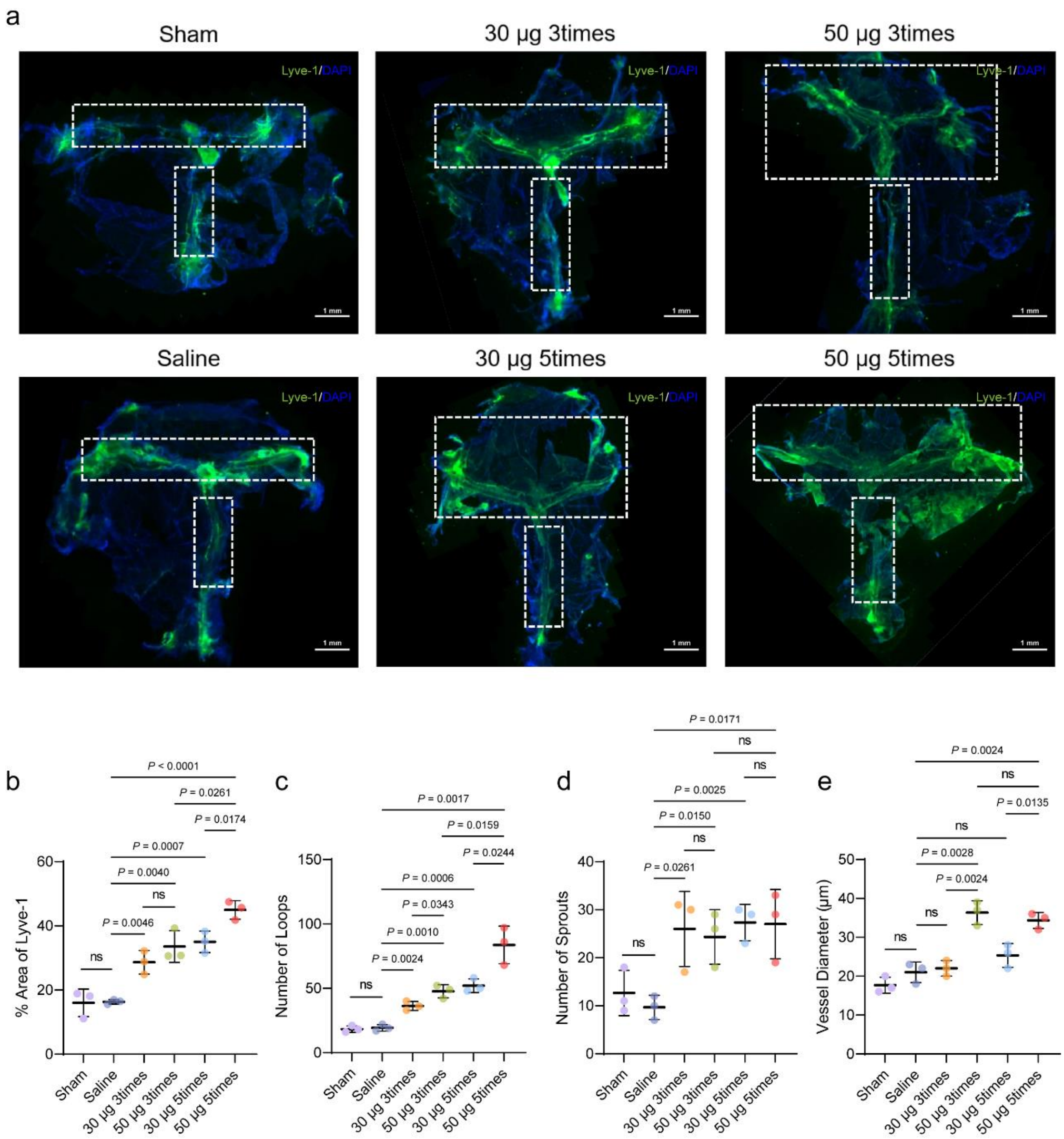

Figure S16. The effect of Nano-plumber on promoting the meningeal lymphangiogenesis using different administration protocols *in vivo*. a) Representative images of meningeal lymphatic vessels (Lyve-1) of using different administration protocols at 28 days after injury (blue: DAPI, green: Lyve-1). b) Quantification of the percent area coverage of Lyve-1 antibody staining (n = 3). c, d) Quantification of the number of (c) loops and (d) sprouts in meningeal whole mounts (n = 3). e) Quantification of the diameters of the meningeal lymphatic vessels. Each data point represents an independent mouse and is an average of 70 measurements along the transverse and superior sagittal sinuses per mouse (n = 3). Data represent mean ± SD of three independent biological replicates. Statistical analyses are performed using one-

way ANOVA with Tukey's post hoc test.

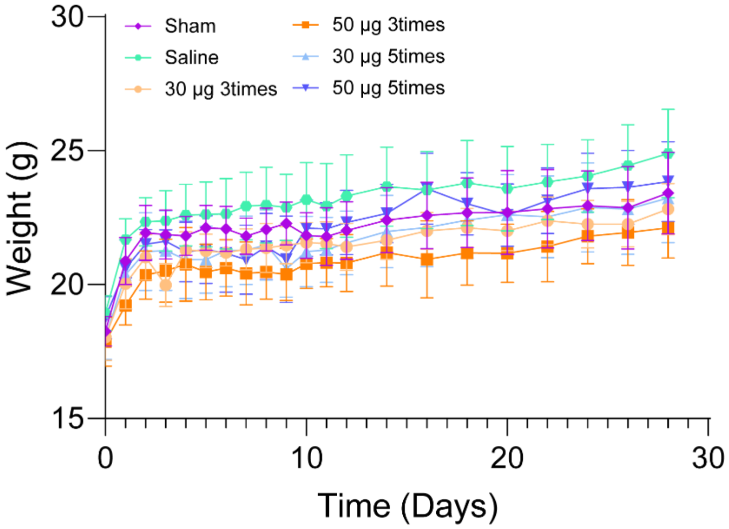

Figure S17. Body weight recorded for different dosage regimens (*n* =6).

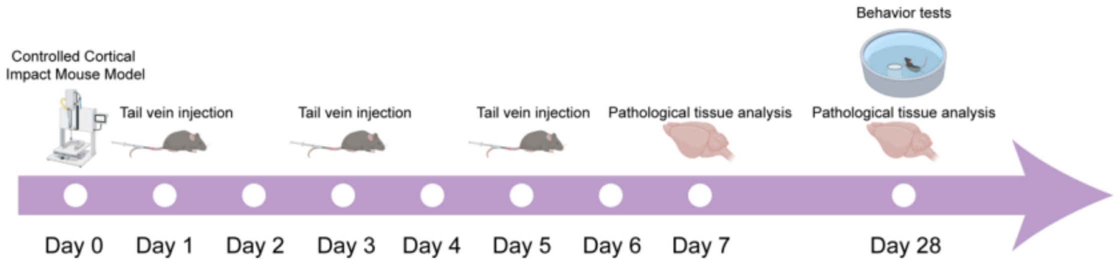

Figure S18. Dosage and testing regimen.

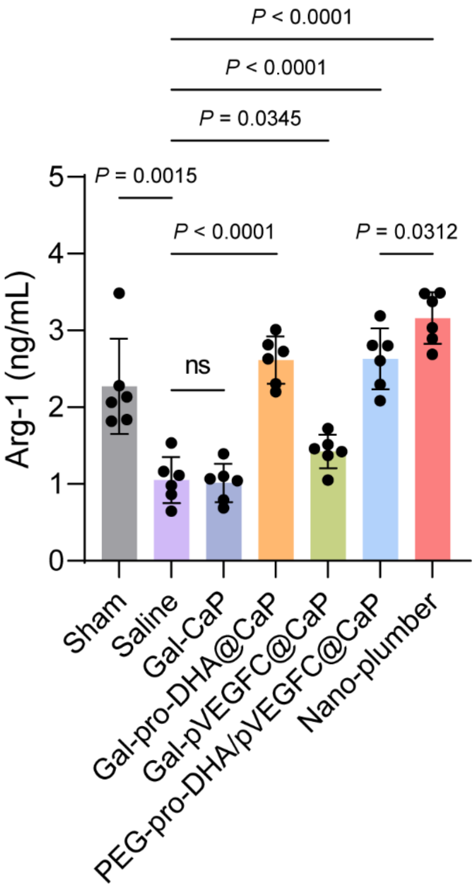

Figure S19. Quantification of the expression level of Arg-1 in the injury brain at 7 days after injury (n = 6). Data represent mean ± SD of six independent biological replicates. Statistical analyses are performed using one-way ANOVA with Tukey's post hoc test.

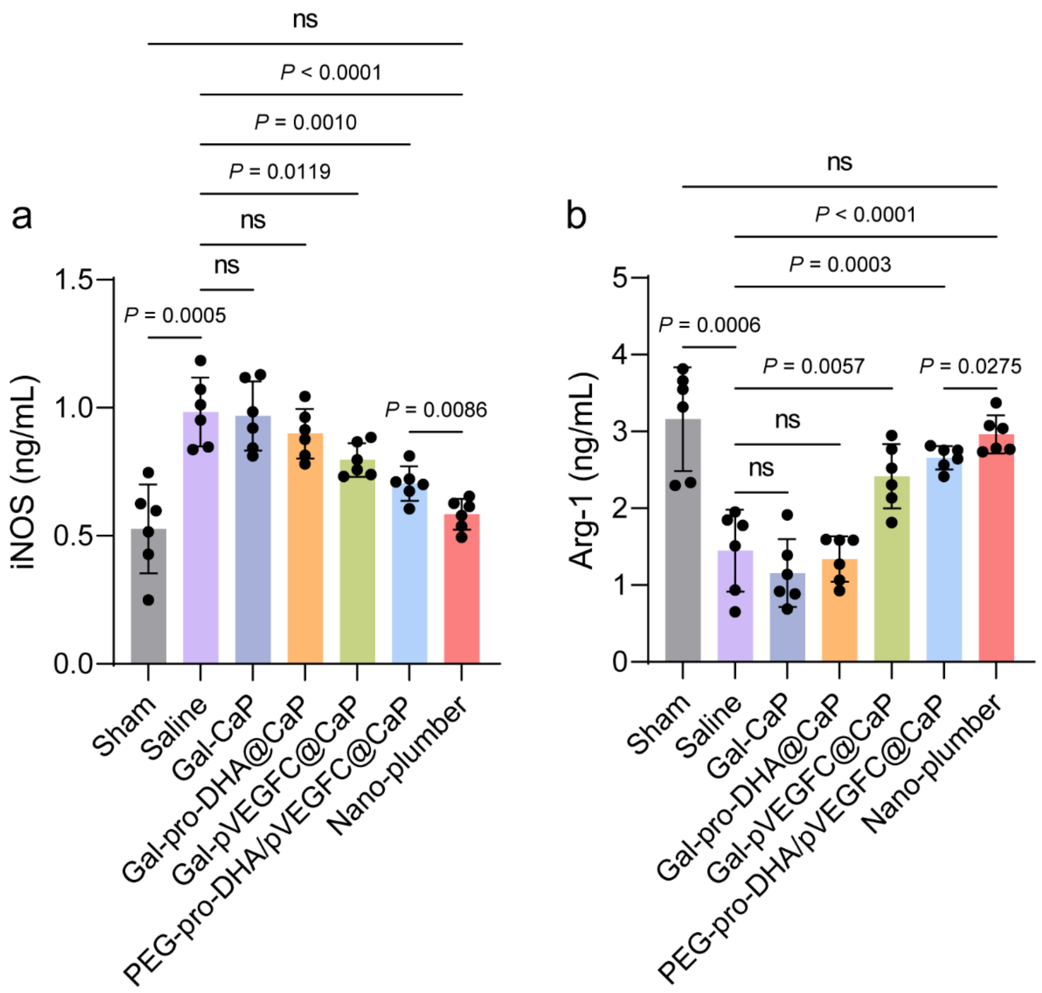

Figure S20. The long-term anti-inflammatory effect of Nano-plumber *in vivo*. a, b) Quantification of the expression level of (a) iNOS and (b) Arg-1 in the brain at 28 days after injury (n = 6). Data represent mean ± SD of six independent biological replicates. Statistical analyses are performed using one-way ANOVA with Tukey's post hoc test.

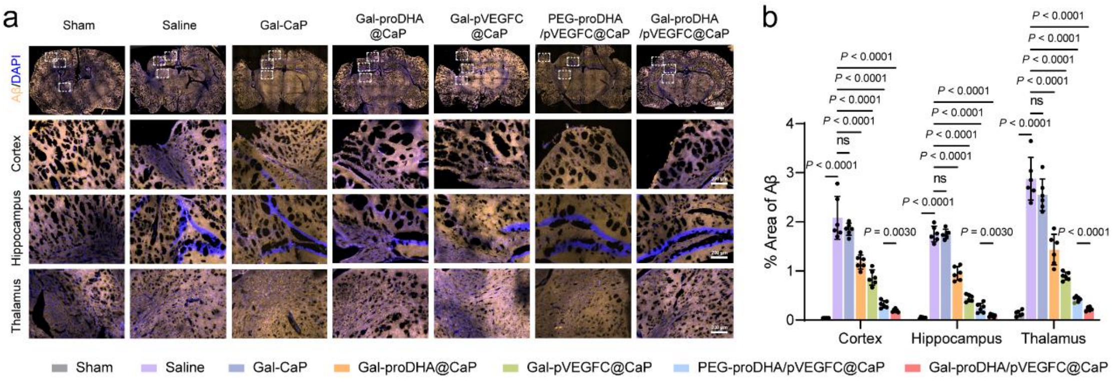

Figure S21. Nano-plumber mitigated comorbid AD after TBI. a, b) Representative

images (a) and quantitative analysis (b) of Aβ burden in injured brain (n = 6). Data represent mean ± SD of three independent biological replicates. Statistical analyses are performed using one-way ANOVA with Tukey's post hoc test.

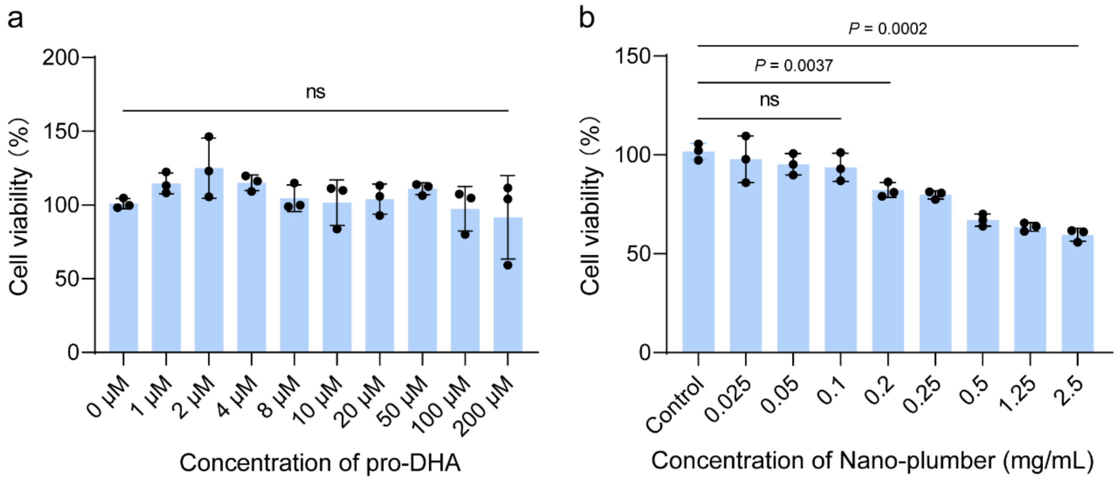

Figure S22. Cytotoxicity assess on BV2 cells. a, b**)** Cytotoxicity assess at different concentrations of (a) pro-DHA or (b) Nano-plumber on BV2 cells (n = 3). Data represent mean ± SD of three independent biological replicates. Statistical analyses are performed using one-way ANOVA with Tukey's post hoc test.

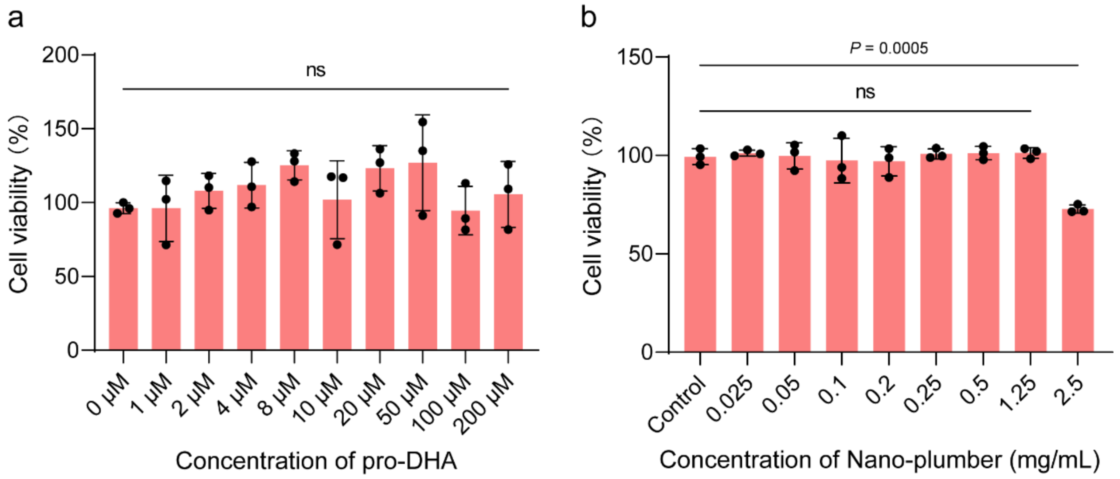

Figure S23. Cytotoxicity assess on HT22 cells. a, b**)** Cytotoxicity assess at different concentrations of (a) pro-DHA or (b) Nano-plumber on HT22 cells (n = 3). Data represent mean ± SD of three independent biological replicates. Statistical analyses are performed using one-way ANOVA with Tukey's post hoc test.

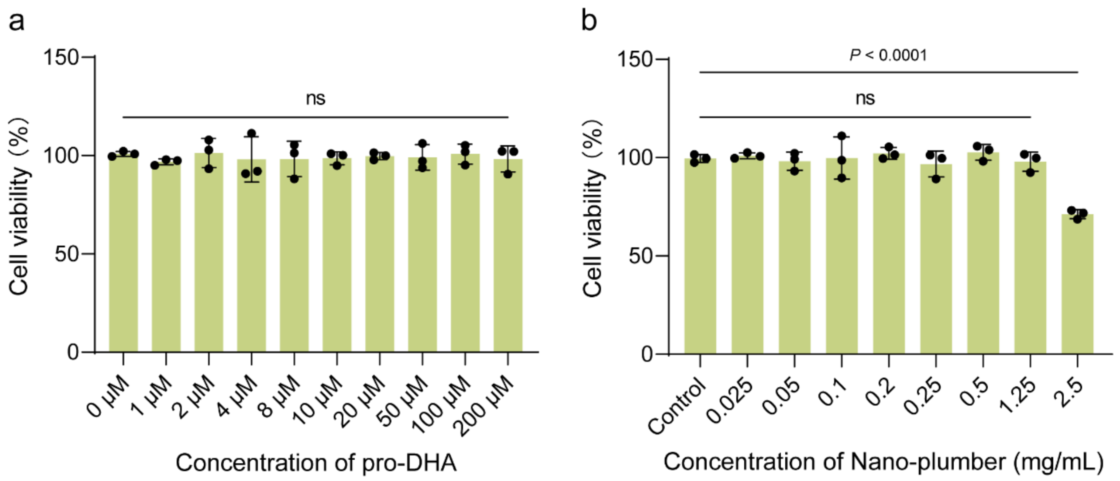

Figure S24. Cytotoxicity assess on bEnd.3 cells. a, b**)** Cytotoxicity assess at different concentrations of (a) pro-DHA or (b) Nano-plumber on bEnd.3 cells (n = 3). Data represent mean ± SD of three independent biological replicates. Statistical analyses are performed using one-way ANOVA with Tukey's post hoc test.

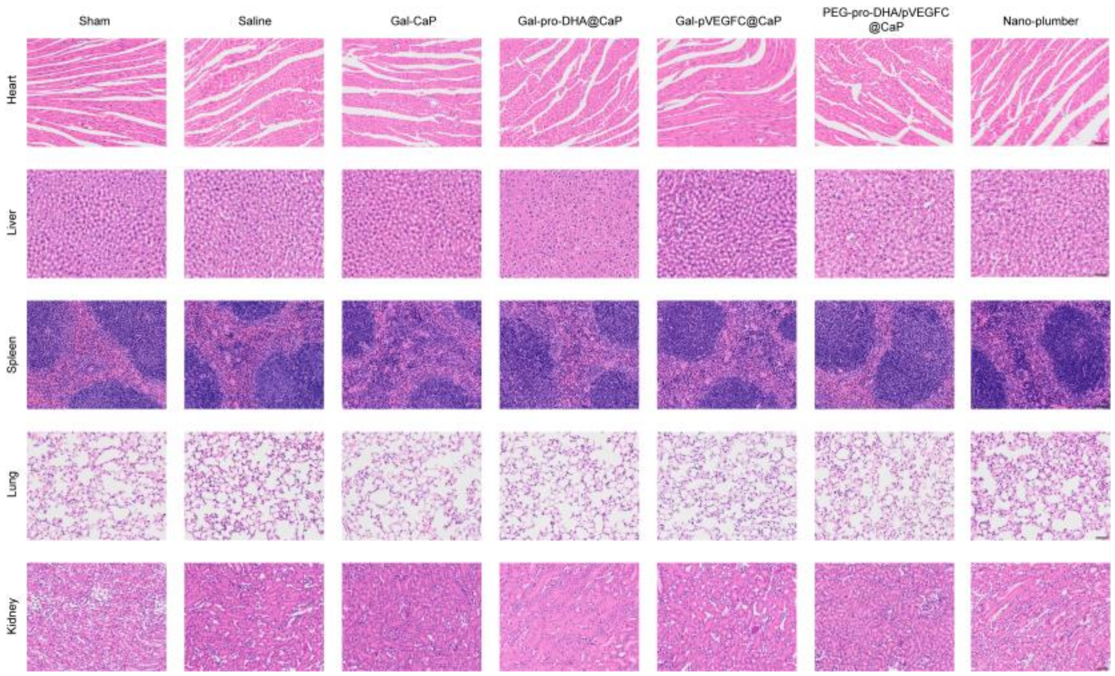

Figure S25. H&E staining to assess the toxicity of various formulations on major organs. Scale bar, 100 μm. The experiments were repeated three times independently.
